# Concise whole blood transcriptional signatures for incipient tuberculosis: a systematic review and patient-level pooled meta-analysis

**DOI:** 10.1016/S2213-2600(19)30282-6

**Published:** 2020-04

**Authors:** Rishi K Gupta, Carolin T Turner, Cristina Venturini, Hanif Esmail, Molebogeng X Rangaka, Andrew Copas, Marc Lipman, Ibrahim Abubakar, Mahdad Noursadeghi

**Affiliations:** a Institute for Global Health, University College London, London, UK; b Division of Infection & Immunity, University College London, London, UK; c Medical Research Council Clinical Trials Unit, University College London, London, UK; d UCL-TB and UCL Respiratory, University College London, London, UK; e Wellcome Centre for Infectious Diseases Research in Africa, Institute of Infectious Diseases and Molecular Medicine, University of Cape Town, Cape Town, South Africa; f Division of Epidemiology and Biostatistics, School of Public Health, University of Cape Town, Cape Town, South Africa; g Department of Respiratory Medicine, Royal Free London NHS Foundation Trust, London, UK

## Abstract

**Background:**

Multiple blood transcriptional signatures have been proposed for identification of active and incipient tuberculosis. We aimed to compare the performance of systematically identified candidate signatures for incipient tuberculosis and to benchmark these against WHO targets.

**Methods:**

We did a systematic review and individual participant data meta-analysis. We searched Medline and Embase for candidate whole blood mRNA signatures discovered with the primary objective of diagnosis of active or incipient tuberculosis, compared with controls who were healthy or had latent tuberculosis infection. We tested the performance of eligible signatures in whole blood transcriptomic datasets, in which sampling before tuberculosis diagnosis was done and time to disease was available. Culture-confirmed and clinically or radiologically diagnosed pulmonary or extrapulmonary tuberculosis cases were included. Non-progressor (individuals who remained tuberculosis-free during follow-up) samples with less than 6 months of follow-up from the date of sample collection were excluded, as were participants with prevalent tuberculosis and those who received preventive therapy. Scores were calculated for candidate signatures for each participant in the pooled dataset. Receiver operating characteristic curves, sensitivities, and specificities were examined using prespecified intervals to tuberculosis (<3 months, <6 months, <1 year, and <2 years) from sample collection. This study is registered with PROSPERO, number CRD42019135618.

**Results:**

We tested 17 candidate mRNA signatures in a pooled dataset from four eligible studies comprising 1126 samples. This dataset included 183 samples from 127 incipient tuberculosis cases in South Africa, Ethiopia, The Gambia, and the UK. Eight signatures (comprising 1–25 transcripts) that predominantly reflect interferon and tumour necrosis factor-inducible gene expression, had equivalent diagnostic accuracy for incipient tuberculosis over a 2-year period with areas under the receiver operating characteristic curves ranging from 0·70 (95% CI 0·64–0·76) to 0·77 (0·71–0·82). The sensitivity of all eight signatures declined with increasing disease-free time interval. Using a threshold derived from two SDs above the mean of uninfected controls to prioritise specificity and positive-predictive value, the eight signatures achieved sensitivities of 24·7–39·9% over 24 months and of 47·1–81·0% over 3 months, with corresponding specificities of more than 90%. Based on pre-test probability of 2%, the eight signatures achieved positive-predictive values ranging from 6·8–9·4% over 24 months and 11·2–14·4% over 3 months. When using biomarker thresholds maximising sensitivity and specificity with equal weighting to both, no signature met the minimum WHO target product profile parameters for incipient tuberculosis biomarkers over a 2-year period.

**Interpretation:**

Blood transcriptional biomarkers reflect short-term risk of tuberculosis and only exceed WHO benchmarks if applied to 3–6-month intervals. Serial testing among carefully selected target groups might be required for optimal implementation of these biomarkers.

**Funding:**

Wellcome Trust and National Institute for Health Research.

## Introduction

Identification of people at high risk of developing tuberculosis enables the delivery of preventive treatment for a disease that accounts for more deaths than any other infectious disease worldwide, with an estimated 10 million incident cases and 1·6 million deaths in 2017.^1^ This approach represents a fundamental component of the WHO End TB strategy, aiming for a 95% reduction in tuberculosis mortality and 90% reduction in tuberculosis incidence by 2035.^2^ However, these efforts are undermined by the poor positive predictive value of available prognostic tests for development of tuberculosis, which focus on the identification of a T-cell-mediated response to mycobacterial antigen stimulation, as a surrogate for latent tuberculosis infection.^3^, ^4^ These tests include the tuberculin skin test and interferon-γ release assays (IGRAs), which have positive predictive values of 1–6% for incident tuberculosis over a 2-year period.^4^, ^5^, ^6^, ^7^ The poor predictive value of available diagnostics precludes precise delivery of preventive therapy, thus increasing costs and potential adverse effects, attenuating the effectiveness of prevention programmes, and reducing roll-out of preventive treatment in limited-resource settings, where most tuberculosis cases occur.

**Research in context**

**Evidence before this study**

We did a systematic review using comprehensive terms for “tuberculosis”, “transcriptome”, “signature” and “blood”, without language or date restrictions, on April 15, 2019. Multiple studies have identified perturbation in the transcriptome that predates clinical diagnosis of tuberculosis and have discovered and assessed performance of one or more signatures for diagnosis of incipient tuberculosis within individual datasets. A head-to-head evaluation of candidate signatures was done, but omitted key signatures, and compared diagnostic accuracy for incipient tuberculosis in only a single dataset over a 0–6-month period. No previous studies have directly compared the diagnostic accuracy of all candidate signatures in a patient-level pooled dataset. It was therefore unknown which signature performs best for diagnosis of incipient tuberculosis, or whether any meets WHO target product profile benchmarks (aiming for sensitivity ≥75% and specificity ≥75% over 2 years).

**Added value of this study**

To our knowledge, we did the largest direct comparison to date of the performance of whole blood transcriptional signatures for diagnosis of incipient tuberculosis. We tested 17 candidate mRNA signatures, identified through a comprehensive systematic review, in a pooled dataset of 1126 RNA sequencing samples from four countries. We show that a single transcript (*BATF2*) and seven other multi-transcript signatures, regulated by interferon signalling, perform with equivalent diagnostic accuracy for incipient tuberculosis. The accuracy of all eight signatures declined markedly with increasing intervals to disease. No signature met the minimum WHO target product profile parameters for incipient tuberculosis biomarkers over a 2-year period. In contrast, the eight best performing signatures met or approximated the minimum target product profile parameters over a 0–3-month period. Using a threshold derived from two SDs above the mean of uninfected controls to prioritise specificity, they achieved sensitivities of 47·1–81·0% and specificities of more than 90%, leading to positive-predictive values of 11·2–14·4% and negative-predictive values of more than 98·9%, when assuming 2% pre-test probability.

**Implications of all the available evidence**

Multiple transcriptional signatures perform with equivalent diagnostic accuracy for incipient tuberculosis. These biomarkers reflect short-term risk of tuberculosis and only exceed WHO benchmarks if applied to 3–6-month intervals. A screening strategy that incorporates serial testing on a 3–6-monthly basis among carefully selected target groups, such as recent case contacts, might be required for optimal implementation of these biomarkers.

Increasing recognition of the continuum of tuberculosis infection and disease has led to renewed interest in the incipient phase of tuberculosis.^8^, ^9^, ^10^ Incipient tuberculosis is defined by WHO as the prolonged asymptomatic phase of early disease before clinical presentation as active disease, during which pathology evolves.^11^ This definition encompasses the incipient and subclinical phases described elsewhere.^12^ Tests that identify the incipient phase, between latent infection and active disease, might lead to improved positive predictive value for incident tuberculosis, while still offering an opportunity to prevent tuberculosis-related morbidity and mortality and reduce onward transmission.^12^ The need for better predictive biomarkers for incident tuberculosis has led to WHO producing a target product profile for incipient tuberculosis diagnostics, stipulating minimum sensitivity and specificity of 75% and optimal sensitivity and specificity of 90% over a 2-year period.^11^ These minimum criteria are based on achieving a positive predictive value of 5·8%, when assuming 2% pre-test probability, to improve on the predictive ability of existing tests.^11^

Multiple studies have shown changes in the host transcriptome in association with active tuberculosis, when compared with healthy controls or individuals with latent tuberculosis infection or other diseases.^13^, ^14^, ^15^, ^16^, ^17^, ^18^, ^19^, ^20^, ^21^, ^22^, ^23^ Signatures have become more concise since the initial discovery of a 393-gene signature of active tuberculosis,^13^ making their translation to near-patient diagnostic tests more achievable. Perturbation in the transcriptome has been found to predate the diagnosis of tuberculosis,^17^, ^24^, ^25^, ^26^ suggesting that transcriptional signatures might offer an opportunity to diagnose incipient tuberculosis and potentially fulfil the WHO target product profile. However, independent validation of each signature is still limited to a small number of datasets. Which of the multiple candidate transcriptional signatures performs best for the identification of incipient tuberculosis or whether any signatures meet the WHO diagnostic accuracy benchmarks remains unclear.

To address these knowledge gaps, we aimed to critically assess the potential value of whole blood transcriptional signatures as biomarkers for incipient tuberculosis in practice.

## Methods

### Search strategy and selection criteria

We hypothesised that any biomarker that distinguishes incipient or active tuberculosis from healthy people might detect incipient disease. We therefore did a systematic review and individual participant data meta-analysis, in accordance with Preferred Reporting Items for a Systematic Review and Meta-analysis of Individual Participant Data standards,^27^ to identify candidate concise whole blood transcriptional signatures for incipient or active tuberculosis and test their diagnostic accuracy for incipient tuberculosis in published whole blood transcriptomic datasets, in which blood sampling and longitudinal follow-up was done. We searched Medline and Embase on April 15, 2019, without language or date restrictions, using comprehensive terms for “tuberculosis”, “transcriptome”, “signature” and “blood”, with screening of identified titles and abstracts done by two independent reviewers. We included candidate whole blood mRNA signatures discovered with a primary objective of diagnosis of active or incipient tuberculosis compared with controls who were either deemed healthy or had latent tuberculosis infection. We tested the performance of eligible signatures in published whole blood transcriptomic datasets where sampling before tuberculosis diagnosis was done and interval time to disease was available. The full search strategy, eligibility criteria, and screening procedures are outlined in appendix 1 (pp 2–3).

In preparation for this meta-analysis, we extended the follow-up of a previously published cohort of London tuberculosis contacts^26^ by relinking the full cohort to national tuberculosis surveillance records (until Dec 31, 2017; median follow-up increased from 0·9 years [IQR 0·7–1·2] to 1·9 years [1·7–2·2]) held at Public Health England using a validated algorithm.^28^ National tuberculosis surveillance records include all statutory national tuberculosis notifications. An additional 27 samples and individuals were also available for inclusion in our analysis. The full updated dataset for this study is available in ArrayExpress (accession number E-MTAB-6845). The London contacts study was approved by the UK National Research Ethics Service (reference 14/EM/1208).^26^ No other ethical approvals were sought for this meta-analysis because all other included patient-level datasets were depersonalised and publicly available.

### Data analysis

Individual-level RNA sequencing data were downloaded for eligible studies and processed (including correction of batch effects) as outlined in appendix 1 (p 3). Only samples obtained before the diagnosis of tuberculosis were included. Prevalent tuberculosis was defined as a tuberculosis diagnosis within 21 days of sample collection, as previously.^4^ Incipient tuberculosis cases were defined as individuals diagnosed with tuberculosis more than 21 days after blood RNA sample collection. Culture-confirmed and clinically or radiologically diagnosed pulmonary or extrapulmonary tuberculosis cases were included in the main analysis. Non-progressors were defined as individuals who remained tuberculosis-free during follow-up. Non-progressor samples with less than 6 months of follow-up from the date of sample collection were excluded owing to risk of outcome misclassification. Participants with prevalent tuberculosis and those who received preventive therapy were excluded. For studies with serial samples from the same individuals, serial samples were included provided that they met these criteria and that they were collected at least 6 months apart, because they were treated as independent samples in the primary analysis.

Scores were calculated for candidate signatures (using the authors' described methods) for each participant in the pooled dataset. For signatures that required reconstruction of support vector machine or random forest models, we validated the reconstructed model against the original authors' model by comparing receiver operating characteristic curves in their original test dataset when possible. Using a predefined control population (including only participants with negative tests for latent tuberculosis infection among the pooled dataset), batch-corrected signature scores were transformed to Z scores (by subtracting the control mean and dividing by SD) to standardise scaling across signatures.^26^

All analyses were done using R (version 3.5.1), unless otherwise specified. Receiver operating characteristic curves for each signature were plotted for a 2-year time horizon. The area under the receiver operating characteristic curve (AUC) and 95% CI were calculated using the DeLong method.^29^ Any data that was originally used to derive specific signatures were excluded from the pooled dataset used to test the performance of the relevant signature. Receiver operating characteristic curves and AUCs for separate study datasets were initially examined to assess the degree of between study heterogeneity. Because little heterogeneity was observed for all signatures, a one-stage individual participant data meta-analysis, assuming common diagnostic accuracy across studies, was done for the primary analysis. AUCs were directly compared in a pairwise approach using paired DeLong tests.^29^ The best performing signature available from all samples in the pooled dataset was used as the reference for comparison with all other signatures; signatures with AUCs smaller than the reference and with p values of less than 0·05 were deemed inferior. Correlation between signature scores was assessed by use of Spearman rank correlation. Pairwise Jaccard similarity indices between signatures were calculated using lists of their constituent genes. Clustered cocorrelation and Jaccard index matrices were generated in Morpheus using average Euclidean distance. Upstream analysis of transcriptional regulation was done using Ingenuity Pathway Analysis (version 49932394) and visualised as network diagrams in Gephi (version 0.9.2), depicting all statistically overrepresented molecules predicted to be upstream of more than two target genes for clarity, to highlight the predicted upstream regulators shared by the constituents of the transcriptional signatures.

Receiver operating characteristic curves and AUCs were assessed for the best performing signatures, using prespecified intervals to tuberculosis from sample collection (<3 months, <6months, <1 year, and <2 years). Sensitivity and specificity for each of these time intervals were determined at predefined cutoffs for each signature, defined as a standardised score of two, representing the 97·7th percentile of the IGRA-negative control population assuming a normal distribution, as in previous work.^26^ These estimates were used to model the estimated predictive values for incident tuberculosis across a range of pre-test probabilities.

We did several sensitivity analyses. First, we restricted inclusion of tuberculosis cases to those with microbiological confirmation. Second, we included only one blood RNA sample per participant from studies that serially sampled by randomly sampling one blood sample per individual. Third, we examined sensitivity and specificity for the best performing signatures using cutoffs defined by the maximal Youden Index^30^ to achieve the highest accuracy within each time interval. Fourth, we recomputed the receiver operating characteristic curves using mutually exclusive time intervals to tuberculosis of 0–3, 3–6, 6–12, and 12–24 months for each curve excluding participants who had developed tuberculosis in an earlier interval. Finally, we did a two-stage individual participant data meta-analysis to ensure consistency with the primary one-stage analysis, as described in appendix 1 (p 3).

This study is registered with PROSPERO, number CRD42019135618.

### Role of the funding source

The funder of the study had no role in study design, data collection, data analysis, data interpretation, or writing of the report. The corresponding author had full access to all the data in the study and had final responsibility for the decision to submit for publication.

## Results

643 unique articles were identified in the systematic review (appendix 1 p 4). Four RNA datasets (table 1) and 17 signatures (table 2) met the criteria for inclusion. The RNA datasets included the Adolescent Cohort Study (ACS) of South African adolescents with latent tuberculosis infection;^24^ the Bill and Melinda Gates Foundation Grand Challenges 6-74 (GC6-74) household tuberculosis contacts study in South Africa, the Gambia, and Ethiopia;^25^ a London tuberculosis contacts study;^26^ and a Leicester tuberculosis contacts study.^17^ All four eligible datasets were publicly available. The ACS and GC6-74 studies were nested case-control designs within larger prospective cohort studies, whereas the London and Leicester tuberculosis contacts studies were prospective cohort studies, with RNA sequencing done for all participants. All four studies were done in HIV-negative participants. The London tuberculosis contacts study included only baseline samples, whereas the ACS, GC6-74, and Leicester tuberculosis contacts studies included serial sampling. All four studies assessed participants for evidence of prevalent tuberculosis at enrolment through clinical evaluation, and the London and Leicester tuberculosis contacts studies also did chest x-rays. The GC6-74 study excluded participants with tuberculosis diagnosed within 3 months of enrolment, and ACS excluded those diagnosed within 6 months. However, participants who developed tuberculosis within these timeframes following serial sampling events were included. All four studies achieved maximal quality assessment scores (appendix 1 pp 5–6).

**Table 1:** Characteristics of the datasets included in meta-analysis of candidate whole blood transcriptional signatures for incipient tuberculosis

	**Samples included**	**Study design**	**Population**	**Setting**	**HIV status**	**Sampling**	**Follow-up duration and method**	**Tuberculosis case definition**	**RNA sequencing methods**	**Newcastle-Ottawa Scale score**	**Baseline tuberculosis assessment**
London tuberculosis contacts^26^	324 (8 tuberculosis; 316 healthy)	Cohort	Adult tuberculosis contacts	London, UK	Negative	Baseline	Median 1·9 (IQR 1·7– 2·2) years, record linkage	Culture-confirmed, or clinically diagnosed	15–20 million 41 bp paired-end reads	7/7	Clinical evaluation and chest x-ray
Adolescent Cohort Study^24^	287 (73 tuberculosis; 214 healthy)	Nested case-control	Adolescents with latent tuberculosis infection	South Africa	Negative	Serial (0, 6, 12, and 24 months)	2 years, active	Intrathoracic disease with 2 positive smears, or 1 positive culture	30 million 50 bp paired-end reads	9/9	Clinical evaluation; tuberculosis <6 months from enrolment excluded; chest x-ray not specified
Grand Challenges 6-74^25^	412 (98 tuberculosis; 314 healthy)	Nested case-control	Adult household pulmonary tuberculosis contacts	South Africa, The Gambia, Ethiopia	Negative	Serial (0, 6, and 18 months)	2 years, active	Culture-confirmed or clinically diagnosed	60 million 50 bp paired-end reads	9/9	Clinical evaluation; tuberculosis <3 months from enrolment excluded; chest x-ray not specified
Leicester tuberculosis contacts^17^	103 (4 tuberculosis; 99 healthy)	Cohort	Adult tuberculosis contacts	Leicester, UK	Negative	Baseline plus serial for a subset*	2 years, active	Confirmed by culture or Xpert MTB/RIF	25 million 75 bp paired-end reads	7/7	Clinical evaluation and chest x-ray

* Owing to the high frequency of serial sampling (<6-monthly), only baseline samples were included.

**Table 2:** Characteristics of candidate whole blood transcriptional signatures for incipient tuberculosis included in systematic review and meta-analysis

	**Original number of genes**	**Model**	**Discovery population**	**Discovery HIV status**	**Discovery setting**	**Discovery approach**	**Intended application**	**Discovery tuberculosis cases**	**Discovery non-tuberculosis controls**	Eligible signatures discovered*
Anderson38^19^†	42	Disease risk score‡	Children	HIV positive and negative	South Africa, Malawi	Elastic net using genome-wide data	Tuberculosis *vs* latent tuberculosis infection	87	43	1
*BATF2*^15^	1	NA	Adults	HIV negative	UK	SVM using genome-wide data	Tuberculosis *vs* healthy (acute *vs* convalescent samples)	46	31	1
Gjoen7^21^	7	LASSO regression§	Children	HIV negative	India	LASSO using 198 preselected genes	Tuberculosis *vs* healthy controls and other diseases	47	36	2
Gliddon3^23^	3	Disease risk score‡	Adults	HIV positive and negative	South Africa, Malawi^16^	Forward Selection-Partial Least Squares using genome-wide data	Tuberculosis *vs* latent tuberculosis infection	285 (tuberculosis and non-tuberculosis)	..	1
Huang11^31^†	13	SVM (linear kernel)	Adults	HIV negative	UK^22^	Common genes from elastic net, L_1/2_ and LASSO models, using genome-wide data	Tuberculosis *vs* healthy controls and other diseases	16	79	1
Kaforou25^16^†	27	Disease risk score‡	Adults	HIV positive and negative	South Africa, Malawi	Elastic net using genome-wide data	Tuberculosis *vs* latent tuberculosis infection	285 (tuberculosis and non-tuberculosis)	..	1
Maertzdorf4^18^	4	Random forest¶	Adults	HIV negative	India	Random forest using 360 selected target genes	Tuberculosis *vs* healthy	113	76	2
NPC2^32^	1	NA	Adults	Not stated	Brazil	Differential expression using genome-wide data	Tuberculosis *vs* healthy	6	28	3
Qian17^33^	17	Sum of standardised expression	Adults	HIV negative	UK^22^	Differential expression of nuclear factor, erythroid 2-like 2-mediated genes	Tuberculosis *vs* healthy controls and other diseases	16	69	1
Rajan5^20^	5	Unsigned sums‡	Adults	HIV positive	Uganda	Differential expression using genome-wide data	Tuberculosis *vs* healthy (active case finding among people living with HIV)	80 total (1:2 cases:controls)	..	1
Roe3^26^	3	SVM (linear kernel)	Adults	HIV negative	UK	Stability selection, using genome-wide data	Incipient tuberculosis *vs* healthy	46	31	1
Singhania20^17^	20	Modified disease risk score‡‖	Adults	HIV negative	UK, South Africa	Random forest using modular approach	Tuberculosis *vs* healthy controls and other diseases	Discovery set not explicitly stated	..	1
Suliman2^7^	2	ANKRD22 – OSBPL10	Adults	HIV negative	Gambia, South Africa, Ethiopia	Pair ratios algorithm using genome-wide data	Incipient tuberculosis *vs* healthy	79	328	4
Suliman4^7^**	4	(GAS6 + SEPT4) –(CD1C + BLK)	Adults	HIV negative	Gambia, South Africa	Pair ratios algorithm using genome-wide data	Incipient tuberculosis *vs* healthy	45	141	4
Sweeney3^14^	3	(GBP5 + DUSP3) ÷ 2 –KLF2	Adults	HIV positive and negative	Meta-analysis	Significance thresholding and forward search in genome-wide data	Tuberculosis *vs* healthy controls and other diseases	266	931	1
Walter45^34^†	51	SVM (linear kernel)	Adults	HIV negative	USA	SVMs, using genome-wide data	Tuberculosis *vs* latent tuberculosis infection	24	24	1
Zak16^24^	16	SVM (linear kernel)	Adolescents	HIV negative	South Africa	SVM-based gene pair models using genome-wide data	Incipient tuberculosis *vs* healthy	37	77	1

Signatures are referred to by combining the first author's name of the corresponding publication as a prefix, with number of constituent genes as a suffix. For signatures where not all constituent genes were identifiable in the RNA sequencing data (eg, due to records being withdrawn), the suffix indicates the number of identifiable genes included in this analysis. Log_2_-transformed transcripts per million data used to calculate all signatures, unless otherwise specified. NA=not applicable. SVM=support vector machine. LASSO=least absolute shrinkage and selection operator.

* Indicates total number of eligible signatures discovered in each study. Where multiple signatures were discovered for the same intended purpose and from the same training dataset, we included the signature with greatest accuracy, as defined by the area under the receiver operating characteristic curve in the validation data. Where accuracy was equivalent, we included the most parsimonious signature.

† Anderson38 included 42 genes in the original, Huang11 had 13, Kaforou25 had 27, and Walter45 had 51 (genes not included in current models were either duplicates or not identifiable in RNA sequencing data).

‡ For disease risk scores, the sum of downregulated genes was subtracted from the sum of upregulated genes. For unsigned sums and modified disease risk scores, genes were summed, irrespective of their direction of regulation.

§ Calculated using non-log-transformed data using model coefficients from original publication.

¶ Required normalisation of the training and test sets. This was done for each gene by subtracting the mean expression across all samples in the dataset and dividing by the SD.

‖ Calculated using non-log-transformed counts per million data with trimmed mean of M-values normalisation, as per original description.

** Modelling approach was not clear from the original description. We recreated this using two approaches: as a simple equation of gene pairs ((GAS6+SEPT4)–(CD1C+BLK)) and as an SVM using the four constituent gene pairs, as previously described.^35^ Because the former approach achieved marginally better performance that was closer to the authors' original description in their test dataset, this was included in the final analysis.

A total of 1126 samples from 905 patients met our criteria for inclusion (appendix 1 p 7). These included 183 samples from 127 incipient tuberculosis cases, of which 117 (92%) were microbiologically confirmed. Eight (6%) of 127 tuberculosis cases were known to be extra-pulmonary, without pulmonary involvement. Baseline characteristics of the study participants are shown in the appendix 1 (pp 8–9). Of note, a large proportion of participants in the London (112 [35%] of 324) and Leicester (86 [83%] of 103) contact studies were of South Asian ethnicity. Principal component analyses revealed clear separation of samples by dataset when including the entire transcriptome, selected genes comprising only the candidate signatures included in the analysis, and invariant genes, indicative of batch effects in the data due to technical variation in RNA sequencing.^36^ These batch effects were eliminated after batch correction (appendix 1 p 10).

Of the 17 identified signatures (table 2), all were discovered from distinct publications, apart from Suliman4 and Suliman2, which were derived from different discovery populations within the same study. Five studies used existing published datasets for discovery,^14^, ^23^, ^26^, ^31^, ^33^ and the remainder used novel data. Two signatures were discovered from paediatric populations.^19^, ^21^ Four signature discovery datasets included HIV-infected and HIV-uninfected participants,^14^, ^16^, ^19^, ^23^ one signature was discovered in an exclusively HIV-infected population for the purpose of active case finding^20^ and the remainder were discovered in HIV-negative populations. Four signatures were discovered with the intention of diagnosis of incipient tuberculosis.^24^, ^25^, ^26^ The remaining 13 were discovered for diagnosis of active tuberculosis disease, of which five^14^, ^17^, ^21^, ^31^, ^33^ targeted discrimination of tuberculosis from other diseases in addition to discriminating people with tuberculosis from people who were healthy or with latent tuberculosis infection. Of the 17 included signatures, only three were not discovered through a genome-wide approach.^18^, ^21^, ^33^ Four signatures required reconstruction of support vector machine models,^24^, ^26^, ^31^, ^34^ and one required reconstruction of a random forest model.^18^ Our reconstructed models were validated against the authors' original descriptions by comparing AUCs in common datasets (appendix 1 p 11). The distribution of signature scores, stratified by study, before and after batch correction is shown in appendix 1 (p 12).

Our analysis initially suggested AUCs for the identification of incipient tuberculosis over a 2-year period were smaller overall in the GC6-74 dataset than in the ACS dataset (appendix 1 pp 13–14). However, the distribution of tuberculosis events during follow-up differed between these studies (appendix 1 pp 8–9). Following stratification by interval to disease, similar AUCs were observed between studies, suggesting that interval to disease confounded the association between source study and AUC. Because little residual between study heterogeneity was observed and principal component analyses after batch correction showed no clustering by study (appendix 1 p 10), we did a pooled data analysis without further adjustment for source study as the primary analysis.

We omitted scores for the Suliman2, Suliman4, and Zak16 signatures for samples comprising their corresponding training sets within the GC6-74 and ACS datasets, but included scores for these signatures for all other samples. The signature with the largest AUC for the identification of incipient tuberculosis over a 2-year period tested in pooled data from all 1126 samples was *BATF2* (AUC 0·74, 95% CI 0·69–0·78). *BATF2* was therefore used as the reference standard for paired comparisons of the other 16 candidate signatures. We found that seven signatures had equivalent AUCs to *BATF2*: Suliman2 (AUC 0·77 [0·71–0·82]), Kaforou25 (0·73 [0·69–0·78]), Gliddon3 (0·73 [0·68–0·77]), Sweeney3 (0·72 [0·68–0·77]), Roe3 (0·72 [0·67–0·77]), Zak16 (0·7 [0·64–0·76]), and Suliman4 (0·7 [0·64–0·76]). The remaining nine signatures had significantly inferior AUCs (appendix 1 p 15). The distributions of the eight best performing signatures among the IGRA-negative control population followed an approximately normal distribution before Z-score transformation (appendix 1 p 16).

The eight signatures identified with equivalent performance showed moderate to high correlation, as defined by Spearman rank correlation (correlation coefficients 0·44–0·84; appendix 1 p 17). In contrast, Singhania20, Anderson38, Huang11, and Walter45 showed little correlation with any other signature. The correlation matrix dendrogram showed the closest associations between signatures identified by the same research group (appendix 1 p 17).

**Figure 1:**
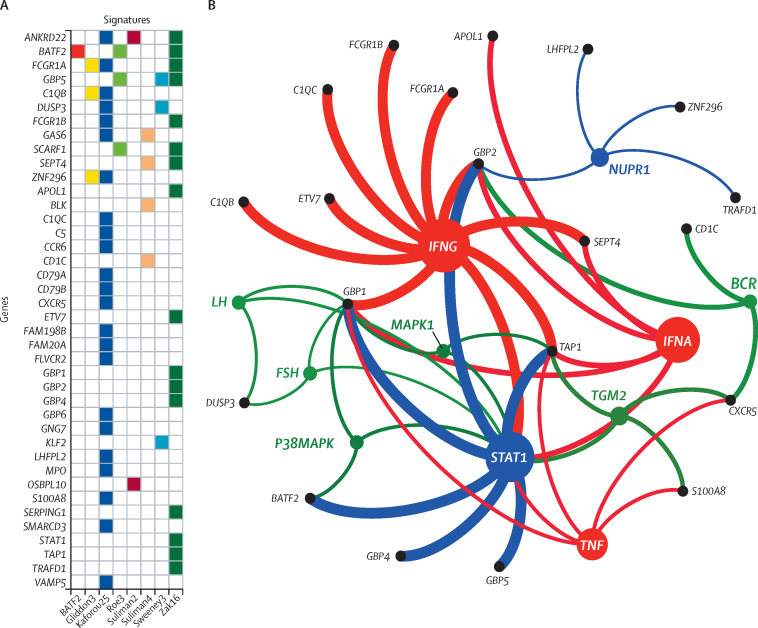
Genes comprising the eight best performing blood transcriptomic signatures for incipient tuberculosis (A) Matrix showing constituent genes for each signature. (B) Network diagram showing statistically enriched (p<0·05) upstream regulators of the 40 genes, identified by Ingenuity Pathway Analysis. Coloured nodes represent the predicted upstream regulators, grouped by function (red=cytokine, blue=transcription factor, green=other). Black nodes represent the transcriptional biomarkers downstream of these regulators. *STAT1*, represented by a blue node as a predicted upstream regulator of a number of genes, is also gene target for other upstream regulators. The identity of each node is indicated using Human Genome Organisation nomenclature. The size of the nodes is proportional to the number of downstream biomarkers associated with each regulator and the thickness of the edges is proportional to the –log_10_ p value for enrichment of each of the upstream regulators.

Spearman rank correlation and Jaccard Index had a weak positive association, suggesting that overlapping constituent genes might partially account for their correlation (appendix 1 p 17). The 40 genes comprising the eight signatures with equivalent AUCs are shown in figure 1A. Upstream analysis predicted that interferon *IFNG, IFNA, STAT1* (the canonical mediator of interferon [IFN] signalling), and tumour necrosis factor (*TNF*) were the strongest predicted transcriptional regulators of these constituent genes (figure 1B; appendix 2).

**Figure 2:**
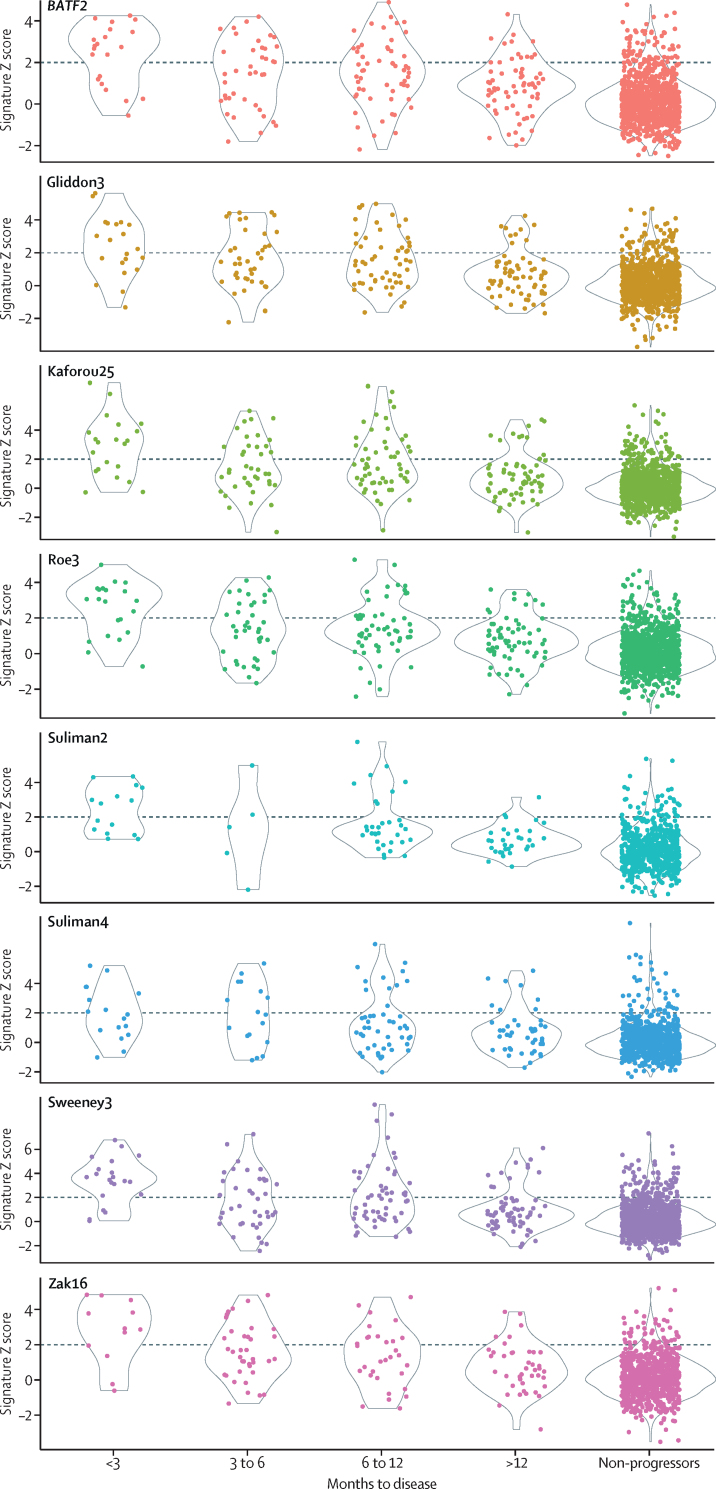
Scatterplots showing scores of eight best performing transcription signatures for incipient tuberculosis, stratified by interval to disease Dashed horizontal lines indicate thresholds set as standardised scores of two for each signature. Number of samples included for each signature, at each timepoint, indicated in the appendix 1 (p 19). Repeated measures analysis of variance with linear trend method showed p<0·0001 for association of categorical interval to disease with decreasing scores for each of the eight signatures. Scatterplots showing scores of these signatures plotted against days to tuberculosis are shown in the appendix 1 (p 18).

**Figure 3:**
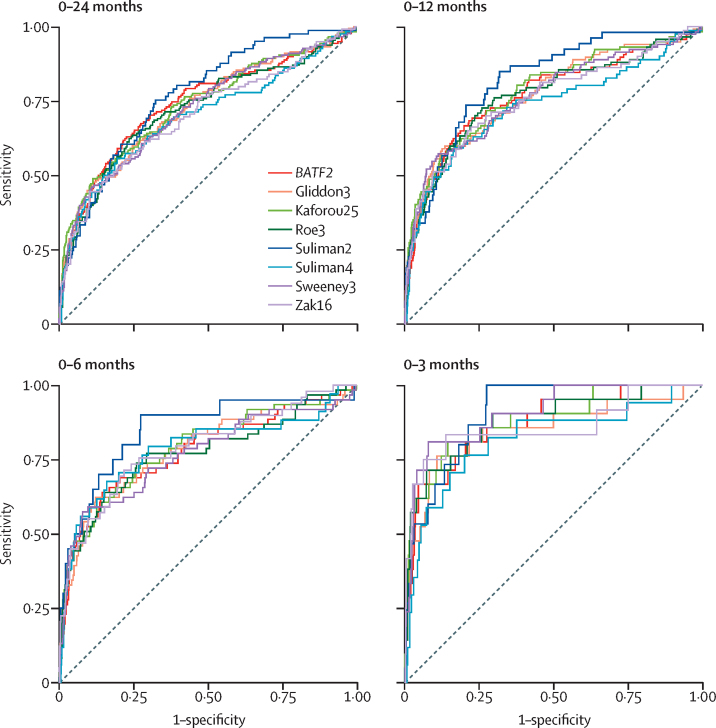
Receiver operating characteristic curves showing diagnostic accuracy of eight best performing transcriptional signatures for incipient tuberculosis Receiver operating characteristic curves shown stratified by months from sample collection to disease. Area under the curve estimates and 95% CIs are shown in the appendix 1 (p 15). Number of samples included for each signature, at each timepoint, indicated in the appendix 1 (p 19).

Scores for the eight best performing signatures, stratified by interval to disease, are shown in figure 2 and appendix 1 (p 18). AUCs of these signatures declined with increasing interval to disease (range 0·82–0·91 for 0–3 months *vs* 0·73–0·82 for 0–12 months; figure 3; appendix 1 p 15).

**Figure 4:**
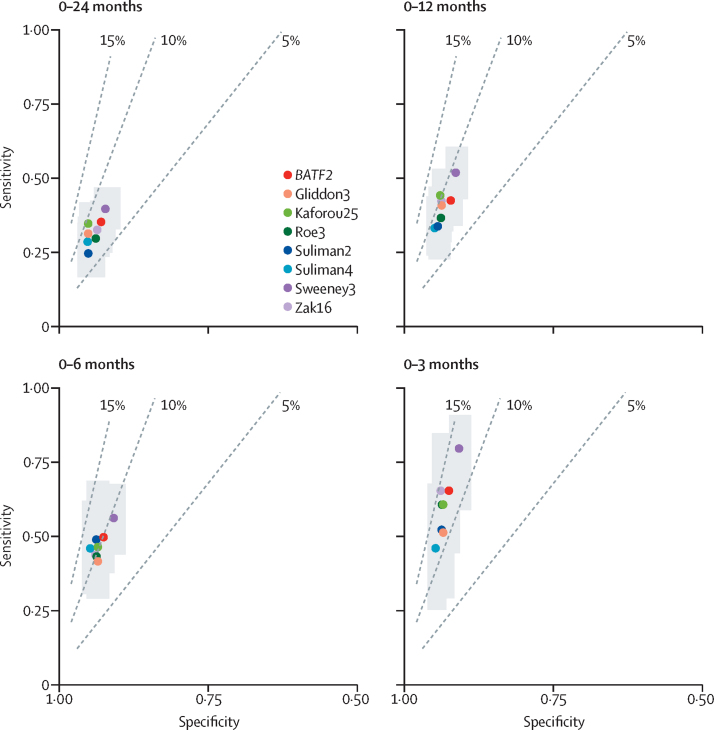
Diagnostic accuracy of eight best performing transcriptional signatures for incipient tuberculosis shown in receiver operating characteristic space, stratified by months to disease Dashed lines represent positive-predictive values of 5%, 10%, and 15%, based on 2% pre-test probability. Grey shading indicates 95% CIs for each signature. Cutoffs derived from two standard scores above the mean of control population. The number of samples included for each signature, at each timepoint, is indicated in the appendix 1 (p 19). Point estimates and 95% CIs are also shown in the appendix 1 (p 20).

Figure 4 shows the diagnostic accuracy of the eight best performing candidates using prespecified cutoffs of standardised score of two based on the 97·7th percentile of the IGRA-negative control population, stratified by interval to disease and benchmarked against positive-predictive value estimates based on a pre-test probability of 2%. At this threshold, test sensitivities over 0–24 months of the eight best performing signatures ranged from 24·7% (95% CI 16·6–35·1) for the Suliman2 signature to 39·9% (33·0–47·2) for Sweeney3, and corresponding specificities ranged from 92·3% (89·8–94·2) to 95·3% (92·3–96·9). In contrast, over a 0–3-month interval, sensitivities ranged from 47·1% (26·2–69·0) for the Suliman4 signature to 81·0% (60·0–92·3) for the Sweeney3 signature, with corresponding specificities of 90·9% (88·9–92·6) to 94·8% (93·0–96·2). For each of the timepoints, the eight signatures had overlapping confidence intervals, and largely fell in the same positive predictive value plane (5–10% over 0–24 months *vs* 10–15% over 0–3 months).

On the basis of a pre-test probability of 2% at the prespecified cutoffs, all eight best performing signatures achieved a positive predictive value marginally above the WHO benchmark of 5·8% for a 0–24-month period, ranging from 6·8% for Suliman2 to 9·4% for Kaforou25, with corresponding negative-predictive values of 98·4% and 98·6% (appendix 1 p 21). For the 0–3-month period, positive predictive values ranged from 11·2% for Gliddon3 to 14·4% for Zak16, with corresponding negative-predictive values of 99·0% and 99·3% (appendix 1 p 21).

Sensitivities and specificities of the eight equivalent signatures using cutoffs defined by the maximal Youden index for each time interval were smaller than the minimum WHO target product profile criteria for a 0–24-month period but met or approximated the minimum criteria over 0–3 months (appendix 1 pp 22–23). Restricting inclusion of incipient tuberculosis cases to those with documented microbiological confirmation and including only one blood RNA sample per participant (by randomly sampling) produced no significant change to the main results (appendix 1 pp 24–25). Reanalysis of the receiver operating characteristic curves using mutually exclusive periods of 0–3, 3–6, 6–12, and 12–24 months magnified the difference in performance between the intervals, with performance declining more markedly with increasing interval to disease (appendix 1 pp 26–27). AUCs in the 12–24-month interval ranged from 0·60 (95% CI 0·50–0·70) to 0·67 (0·60–0·75) for the eight equivalent signatures. Finally, our two-stage meta-analysis approach showed similar findings to the primary analysis (appendix 1 pp 28–29).

## Discussion

To our knowledge, this is the largest analysis to date of the performance of whole blood transcriptional signatures for incipient tuberculosis. We showed that eight candidate signatures performed with equivalent diagnostic accuracy over a 2-year period. These signatures ranged from a single transcript (*BATF2*) to 25 genes (Kaforou25). The accuracy of all eight signatures declined markedly with increasing intervals to disease. These signatures only marginally surpassed the WHO target positive predictive value of 5·8% over 2 years, assuming 2% pre-test probability and using a cutoff of two standard scores. However, sensitivity at this cutoff was only 24·7–39·9%, missing most cases. No signature achieved the WHO target sensitivity and specificity of 75% or more over 2 years, even when using the cutoff with maximal accuracy. In contrast, using two standard scores cutoffs over a 0–3-month period, the eight best performing signatures achieved sensitivities of 47·1–81·0% and specificities of more than 90%. This led to positive-predictive values of 11·2–14·4% and negative-predictive values of more than 98·9%, when assuming 2% pre-test probability, suggesting that the WHO target product profile can be achieved over shorter time intervals.

To achieve the WHO target product profile, a screening strategy that incorporates serial testing on a 3–6-monthly basis might therefore be required for transcriptional signatures. Such a strategy, however, is unlikely to be feasible at a population level. Instead, high-risk groups, such as household contacts, could be targeted. However, even this approach will be challenging in high-transmission settings, given the limited global coverage of contact-tracing programmes. In low-transmission, high-resource settings, serial blood transcriptional testing for risk stratification over a defined 1–2-year period might be more achievable, particularly among recent contacts or new entry migrants from high-transmission countries, for whom risk of disease is highest within an initial 2 year interval.^4^, ^26^, ^37^ Integral to scale-up of the use of these biomarkers is translation of transcriptional measurements from genome-wide approaches to the reproducible quantification of selected signature gene transcripts, with appropriately defined cutoffs. Although such targeted transcript quantification has been done for some signatures using PCR-based platforms,^23^, ^24^, ^25^, ^38^ no signature platforms have been validated for implementation in a near-patient or commercial assay. An additional challenge to implementation is the cost of these assays. This cost is likely to far exceed the US$2 target specified by the WHO target product profile for a nonsputum triage test for tuberculosis disease,^39^ but might achieve the WHO target price to identify incipient tuberculosis for less than $100, using the price of IGRAs as an initial benchmark.^11^ The fact that a number of different signatures show equivalent performance enables greater freedom for commercial development of this approach by overcoming restricted access to specific signatures protected by intellectual property rights and encouraging competition to drive down costs.

The eight signatures that achieved equivalent performance were discovered with the primary intention of diagnosis of incipient tuberculosis,^24^, ^25^, ^26^ or differentiating active tuberculosis from people who are healthy or with latent tuberculosis infection.^14^, ^15^, ^16^, ^23^ Discovery populations for these eight signatures included adults or adolescents from the UK or sub-Saharan Africa,^15^, ^16^, ^23^, ^24^, ^25^, ^26^ or a meta-analysis of microarray data from multiple studies,^14^ including a minimum of 37 incipient or active tuberculosis cases. All eight signatures were discovered using genome-wide approaches. In contrast, the nine signatures with inferior performance included two derived from studies in children,^19^, ^21^ one from a study that prioritised discrimination of active tuberculosis from other bacterial and viral infections,^17^ and one from a study that conducted active case-finding for tuberculosis among people living with HIV.^20^ The differences in primary intended applications, which are reflected in the study populations used for biomarker discovery, might account for their inferior performance when evaluated solely for identification of incipient tuberculosis in a predominantly healthy, HIV-negative adult and adolescent population. The signatures with inferior performance also included three discovered in panels of pre-selected candidate genes, rather than a genome-wide approach,^18^, ^21^, ^33^ and four with only 6–24 tuberculosis cases in the discovery sets.^31^, ^32^, ^33^, ^34^ These observations suggest that use of a genome-wide approach and inclusion of adequate numbers of diseased cases should be considered during signature discovery to increase the likelihood of identifying generalisable signatures.

The eight best performing signatures were derived from the application of different computational approaches but showed moderate to high levels of cocorrelation, with the closest associations between signatures identified by the same research group. This finding likely reflects common discovery datasets and modelling approaches used within research groups. Overlapping constituent genes only partially accounted for correlation between signatures, suggesting that they reflect different dimensions of a common host response to infection with *Mycobacterium tuberculosis*. This hypothesis was strongly supported by the identification IFN and TNF signalling pathways as statistically enriched upstream regulators of the genes across the eight signatures. Although these host response pathways are unlikely to be specific to tuberculosis, the application of these biomarkers for incipient tuberculosis mitigates against the limitations of imperfect specificity by focusing on asymptomatic individuals in whom the probability of other diseases is low. The time-dependent sensitivity of the signatures suggests that the duration of the incipient phase of tuberculosis is typically 3–6 months. However, even within the less than 3-month time interval, the sensitivity of the best performing transcriptional signatures ranged from 47·1–81·0%, indicating that the biomarkers might have imperfect sensitivity for incipient tuberculosis or that the incipient phase can progress very rapidly among a subset of cases. Each signature did exhibit an AUC of more than 0·5 for discriminating incipient tuberculosis from non-progressors even 12–24 months after sampling, suggesting that the incipient phase might be more prolonged in some cases. These slowly progressive cases might reflect those in which the host response initially achieves mycobacterial control in dynamic host–pathogen interactions.^40^ These findings are generally mirrored in proteomic and metabolomic data from similar cohorts.^41^, ^42^

The strengths of this study include the size of the pooled dataset, including 1126 samples from 905 patients and 183 samples from 127 incipient tuberculosis cases. Individual-level data were available for all four eligible studies, all of which achieved maximal quality assessment scores and were done in relevant target populations of either recent tuberculosis contacts or people with latent tuberculosis infection. This facilitated a robust analysis of the diagnostic accuracy of the candidate signatures, stratified by interval to disease. Additionally, we did a comprehensive systematic review and identified 17 candidate signatures. For each of these signatures, gene lists and modelling approaches were extracted and validated by independent reviewers. Moreover, for signatures that required model reconstruction, our models were cross-validated against original models by comparing AUCs using the same dataset wherever possible. This approach facilitated a comprehensive, head-to-head analysis of candidate signatures for incipient tuberculosis for the first time, ensuring that each head-to-head comparison was done on paired data. This approach contrasts with a head-to-head systematic evaluation that included only two of the eight best-performing signatures in our analysis and compared performance for incipient tuberculosis in only one dataset over a 0–6-month period.^35^ Furthermore, our meta-analytic methods ensured a standardised approach to RNA sequencing data, which included an unbiased approach to batch correction, with unchanged distributions of signature scores within each dataset following correction.

A weakness of our analysis is that we were unable to do subgroup analyses by age, ethnicity, or country, because the contributing studies largely defined these strata. There were no clear differences in performance by study, supporting the generalisability of the results. We were also unable to account for previous BCG vaccination status, although we anticipate that BCG coverage is likely to be very high among the study populations included. Additionally, having observed little heterogeneity between studies, we did a pooled analysis, assuming common diagnostic accuracy between studies. The precision of our estimates therefore might be slightly overstated and statistical tests might be anti-conservative. However, sensitivity analysis using a two-stage meta-analysis approach with random effects yielded similar findings, supporting the robustness of our results. Likewise, treating serial samples as independent was anti-conservative, but findings were similar in our sensitivity analysis taking only one sample per individual at random.

All included datasets were from sub-Saharan Africa and the UK, although a substantial proportion of Asian participants were included in the UK studies. No data were available for people living with HIV or children younger than 10 years, among whom different blood transcriptional perturbations might occur in tuberculosis.^8^, ^19^ Prospective validation studies in other regions and among these specific target populations are needed and could be used to periodically update this meta-analysis to further increase generalisability. Only eight tuberculosis cases were known to be extra-pulmonary, thus precluding assessment of diagnostic accuracy stratified by tuberculosis disease site. Additionally, most incipient tuberculosis cases were contributed from the African datasets, with 12 cases from the UK studies. Nevertheless, the UK studies were done in appropriate target populations of close contacts of tuberculosis index cases and were done as cohort studies, as opposed to the African case-control designs. High specificity for correctly identifying non-progressors among contacts is a key attribute in improving positive predictive value compared with existing tests. Hence, these UK datasets were valuable additions to the pooled meta-analysis. Furthermore, when multiple signatures were discovered from the same discovery population and for the same purpose, we only included the best performing signature from the original study's validation set in our analysis. We therefore excluded a small number of worse-performing candidate signatures to prioritise a parsimonious list of the most promising candidates. The probability of these excluded signatures performing better than the included signatures is therefore negligible.

In summary, we show for the first time that eight transcriptional signatures, including a single transcript (*BATF2*), have equivalent diagnostic accuracy for identification of incipient tuberculosis. Performance appeared similar across studies, including participants from the UK and sub-Saharan Africa. Signature performance was highly time-dependent, with lower accuracy at longer intervals to disease. A screening strategy that incorporates serial testing on a 3–6-monthly basis among selected high-risk groups might be required for these biomarkers to surpass WHO target product profile benchmarks.
