# Supplementary material for: Concise whole blood transcriptional signatures for incipient tuberculosis: a systematic review and patient-level pooled meta-analysis
Source: Lancet Respir Med. 2020 Apr;8(4):395–406. doi: 10.1016/S2213-2600(19)30282-6 (PMC7113839; doi:10.1016/S2213-2600(19)30282-6)

THE LANCET
Respiratory Medicine

Supplementary appendix 1

This appendix formed part of the original submission and has been peer reviewed. We post it as supplied by the authors.

Supplement to: Gupta RK, Turner CT, Venturini C, et al. Concise whole blood transcriptional signatures for incipient tuberculosis: a systematic review and patient-level pooled meta-analysis. *Lancet Respir Med* 2020; published online Jan 17. https://doi.org/10.1016/S2213-2600(19)30282-6.

Concise whole blood transcriptional signatures for incipient tuberculosis: A systematic review and patient-level pooled meta-analysis

Supplementary Appendix

Supplementary Methods

Details of search strategy

We searched Medline and Embase on 15/04/2019, with no language or date restrictions, using comprehensive MeSH and keyword terms for ‘biomarkers’ (terms 1-11 below); ‘tuberculosis (12); ‘transcriptome’ (13-19); and ‘blood’ (20-22). We consolidated the search by also hand-searching reference lists of relevant review articles. The Medline search strategy is outlined below (mp = ‘multi-purpose’ term for Medline key word searches).

1. Biomarkers/
2. Diagnostic Tests, Routine/
3. "Predictive Value of Tests"/
4. diagnostic test*.mp.
5. biomarker*.mp.
6. ppv.mp.
7. npv.mp.
8. sensitivit*.mp.
9. specificit*.mp.
10. signature*.mp.
11. 1 or 2 or 3 or 4 or 5 or 6 or 7 or 8 or 9 or 10
12. exp TUBERCULOSIS/ or tuberculosis.mp. or exp MYCOBACTERIUM TUBERCULOSIS/ or tb.mp.
13. RNA/
14. Transcriptome/
15. rna.mp.
16. transcript*.mp.
17. gene expression.mp.
18. Gene Expression Profiling/ or RNA, Messenger/ or Transcription, Genetic/ or Gene Expression/
19. 13 or 14 or 15 or 16 or 17 or 18
20. blood/
21. blood.mp.
22. 20 or 21
23. 11 and 12 and 19 and 22
24. remove duplicates from 23
25. limit 24 to "humans only (removes records about animals)"

Eligibility criteria for candidate signatures

We included concise whole blood mRNA signatures discovered with a primary objective of diagnosis of active or incipient TB, compared to controls who were either deemed healthy, or had latent TB infection. In the absence of a standardised definition, we defined ‘concise’ as signatures that used a defined approach to feature selection to reduce multidimensionality and the number of constituent genes, thus leading to biomarkers that may be more amenable to clinical translation. The availability of gene names that comprise the signature, along with the corresponding equation or modelling approach was required. We also specified that the signature (including component genes, and modelling approach) was validated in at least one independent test or validation set, in order to enable reliable signature reconstruction. We only included signatures discovered from training sets that included controls who were either deemed healthy, or had latent TB infection, since discriminating incipient TB from healthy or latently infected people is the primary aim of incipient TB diagnostics. Where multiple signatures were discovered for the same intended purpose and from the same training dataset, we included the signature with greatest accuracy (as defined by the area under the receiver operating characteristic curve (AUROC) in the validation data) in order to prioritise a parsimonious list of the most promising candidate signatures. Where accuracy was equivalent, we included the signature with fewest number of genes.

Eligibility criteria for transcriptomic datasets

We included published whole blood transcriptomic datasets (RNAseq or microarray) where sampling prior to TB diagnosis was performed and interval time to disease was available. We specified a minimum median duration of follow-up of one year to reduce the risk of outcome misclassification. For studies where preventative TB therapy was offered, individual level data was required to identify the treated cases.

Screening and data extraction

Two independent reviewers (RKG and CTT) screened titles and abstracts identified in the search, and determined eligibility for final inclusion following full-text review. Gene lists and corresponding equations or modelling approaches were extracted for each eligible candidate signature and checked by a second reviewer. Disagreements regarding study inclusion or signature calculations were resolved by a third reviewer (MN). Quality assessment and risk of bias were assessed for the studies corresponding to included RNA datasets, using modified versions of the Newcastle-Ottowa scale (using the cohort or case-control version as appropriate to each contributing study)[1].

RNA data processing

Individual level RNAseq data were downloaded for eligible studies, and mapped to the reference transcriptome (Ensembl Human GRCh38 release 95) using Kallisto[2]. The transcript-level output counts and transcripts per million (TPM) values were summed on gene level and annotated with gene symbols using tximport and BioMart[3,4]. Only protein-coding genes were selected for downstream processing, and TPM and counts per million (CPM) values <0.001 were set to 0.001 prior to log2 transformation. TPM data were compared for participants from different studies using principal component analysis (PCA) to test for heterogeneity and determine the need for batch correction. This included (a) the entire transcriptome; (b) selected genes comprising only the candidate signatures included in the analysis; and (c) invariant genes that were in the lowest quartile of genes ranked by variance within each of the contributing datasets. Batch correction was performed using the COmbat CO-Normalization Using conTrols (COCONUT) package in R[5]. This approach facilitated correction based on the disease-free controls, which was then applied to those with disease, thus reducing risk of bias during correction due to differing prevalence of disease among the study populations included.

Sensitivity analysis: two stage individual participant data meta-analysis (IPD-MA)

We performed a one-stage IPD-MA in the primary analysis. In this sensitivity analysis, we conducted a two-stage IPD-MA. To do this, we calculated AUCs for each signature, stratified by interval to disease, in each contributing dataset separately, prior to batch correction. We then derived pooled AUCs and 95% confidence intervals for each signature across studies using random-effects meta-analysis of logit-transformed AUCs, using the *metamisc* package in R[6]. We also calculated sensitivity, specificity and predictive values at Z2 score cut-offs for each signature within each batch-corrected dataset, and derived pooled estimates using bivariate random-effects meta-analysis in the *mada* package in R[7].

References

1 Wells G, Shea B, O’Connell D, *et al.* The Newcastle-Ottawa Scale (NOS) for assessing the quality of nonrandomised studies in meta-analyses. http://www.ohri.ca/programs/clinical_epidemiology/oxford.asp (accessed June 3, 2019).

2 Bray NL, Pimentel H, Melsted P, Pachter L. Near-optimal probabilistic RNA-seq quantification. *Nat Biotechnol* 2016; **34**: 525–7.

3 Soneson C, Love MI, Robinson MD. Differential analyses for RNA-seq: transcript-level estimates improve gene-level inferences. *F1000Research* 2015; **4**: 1521.

4 Smedley D, Haider S, Durinck S, *et al.* The BioMart community portal: an innovative alternative to large, centralized data repositories. *Nucleic Acids Res* 2015; **43**: W589–98.

5 Sweeney TE, Wong HR, Khatri P. Robust classification of bacterial and viral infections via integrated host gene expression diagnostics. *Sci Transl Med* 2016; **8**: 346ra91-346ra91.

6 Snell KI, Ensor J, Debray TP, Moons KG, Riley RD. Meta-analysis of prediction model performance across multiple studies: Which scale helps ensure between-study normality for the *C* -statistic and calibration measures? *Stat Methods Med Res* 2018; **27**: 3505–22.

7 Reitsma JB, Glas AS, Rutjes AWS, Scholten RJPM, Bossuyt PM, Zwinderman AH. Bivariate analysis of sensitivity and specificity produces informative summary measures in diagnostic reviews. *J Clin Epidemiol* 2005; **58**: 982–90.

Supplementary Figure 1. Flowchart showing systematic review process for review and meta-analysis of concise whole blood transcriptional signatures for incipient tuberculosis. LTBI = latent TB infection.

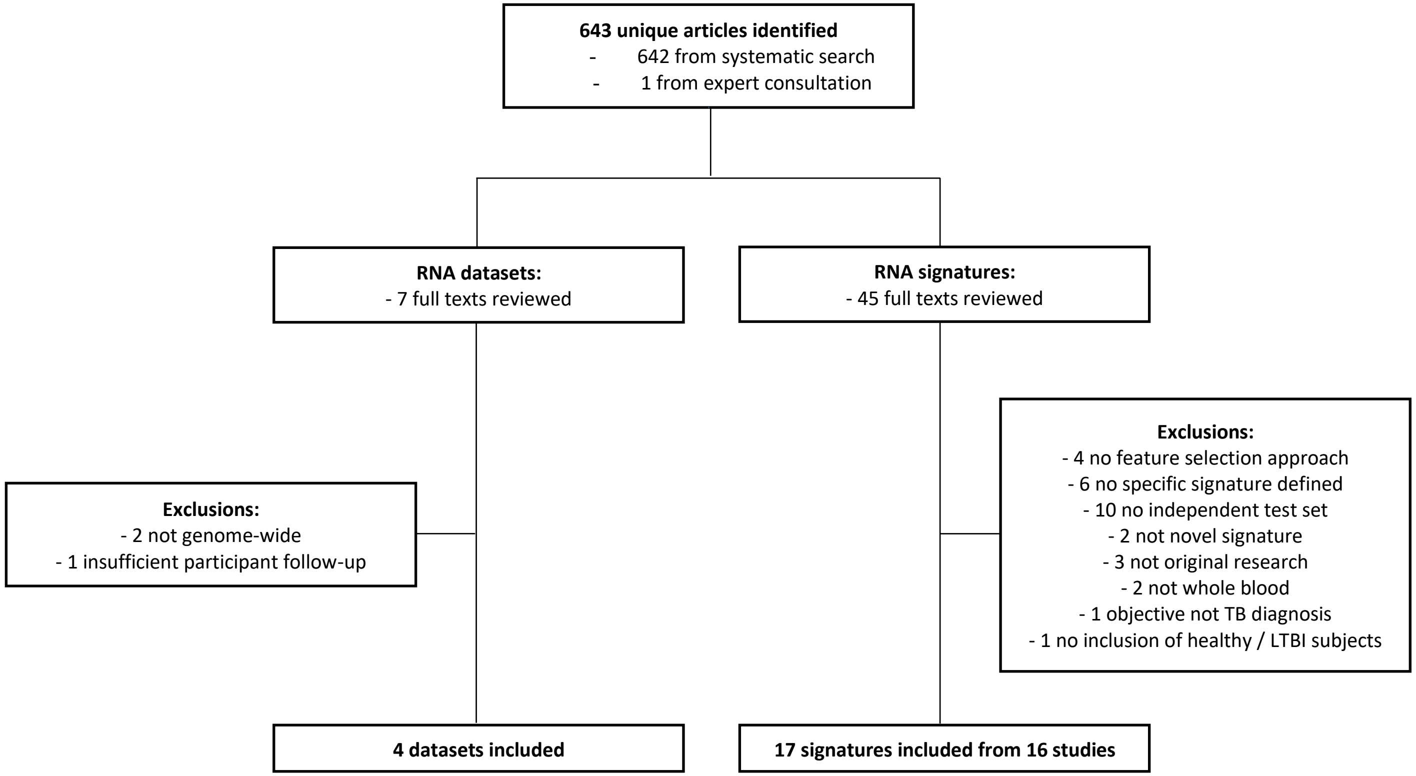

Supplementary Table 1. Quality assessment of four studies representing datasets included in meta-analysis, using Newcastle-Ottowa scale for (a) case-control studies; or (b) cohort studies, as appropriate. ACS = adolescent cohort study; GC6-74 = Bill and Melinda Gates Foundation Grand Challenges 6-74 TB contacts study.

(a)

**Case-control studies**	**GC6-74**	**Comments**	**ACS**	**Comments**
**Selection**				
1) Is the case definition adequate?				
a) yes, with independent validation Ø	✓	Detailed data available	✓	Microbiologically confirmed only
b) yes, eg record linkage or based on self reports				
c) no description				
2) Representativeness of the cases				
a) consecutive or obviously representative series of cases Ø	✓		✓	
b) potential for selection biases or not stated				
3) Selection of Controls				
a) community controls Ø	✓		✓	
b) hospital controls				
c) no description				
4) Definition of Controls				
a) no history of disease (endpoint) Ø	✓		✓	
b) no description of source				
**Comparability**				
1) Comparability of cases and controls on the basis of the design or analysis				
a) study controls for age Ø	✓		✓	
b) study controls for any additional factor Ø	✓	Recruitment region, sex, enrolment year	✓	Gender, ethnicity, school, previous TB
**Exposure**				
1) Ascertainment of exposure	✓	Raw RNAseq data available	✓	Raw RNAseq data available
a) secure record (eg surgical records) Ø				
b) structured interview where blind to case/control status Ø				
c) interview not blinded to case/control status				
d) written self report or medical record only				
e) no description				
2) Same method of ascertainment for cases and controls				
a) yes Ø	✓	Calculated using RNAseq data	✓	Calculated using RNAseq data
b) no				
3) Non-Response rate				
a) same rate for both groups Ø	✓		✓	
b) non respondents described				
c) rate different and no designation				
**Total (max 9)**	**9**		**9**	

(b)

Selection	London Contacts	Comments	Leicester Contacts	Comments
1) Representativeness of the exposed cohort				
a) truly representative of the average TB contact in the community Ø	✓		✓	
b) somewhat representative of the average TB contact in the community Ø				
c) selected group of users eg nurses, volunteers				
d) no description of the derivation of the cohort				
2) Selection of the non exposed cohort				
a) drawn from the same community as the exposed cohort Ø	✓	Based on RNAseq data	✓	Based on RNAseq data
b) drawn from a different source				
c) no description of the derivation of the non exposed cohort				
3) Ascertainment of exposure				
a) secure record (eg surgical records) Ø	✓	Based on RNAseq data	✓	Based on RNAseq data
b) structured interview Ø				
c) written self report				
d) no description				
4) Demonstration that outcome of interest was not present at start of study				
a) yes Ø	✓		✓	
b) no				
**Comparability**				
1) Comparability of cohorts on the basis of the design or analysis	N/A		N/A	
**Outcome**				
1) Assessment of outcome				
a) independent blind assessment Ø			✓	
b) record linkage Ø	✓			
c) self report				
d) no description				
2) Was follow-up long enough for outcomes to occur				
a) yes (>1 year) Ø	✓	Median 1.9 years	✓	2 years (clarified with authors)
b) no				
3) Adequacy of follow up of cohorts				
a) complete follow up - all subjects accounted for Ø	✓	Validated linkage method	✓	Clarified with authors
b) subjects lost to follow up unlikely to introduce bias - small number lost, or description provided of those lost) Ø				
c) follow up rate < 80% and no description of those lost				
d) no statement				
**Total (max 7)**	**7**		**7**	

Supplementary Figure 2. Inclusion of samples from contributing datasets in meta-analysis of concise whole blood transcriptional signatures for incipient tuberculosis (TB). ACS = adolescent cohort study; GC6-74 = Bill and Melinda Gates Foundation Grand Challenges 6-74 TB contacts study. [$]Prevalent TB defined as TB diagnosed within 21 days of sample collection. *Indicates >1 sample collected from the same participant within a 6-month interval.

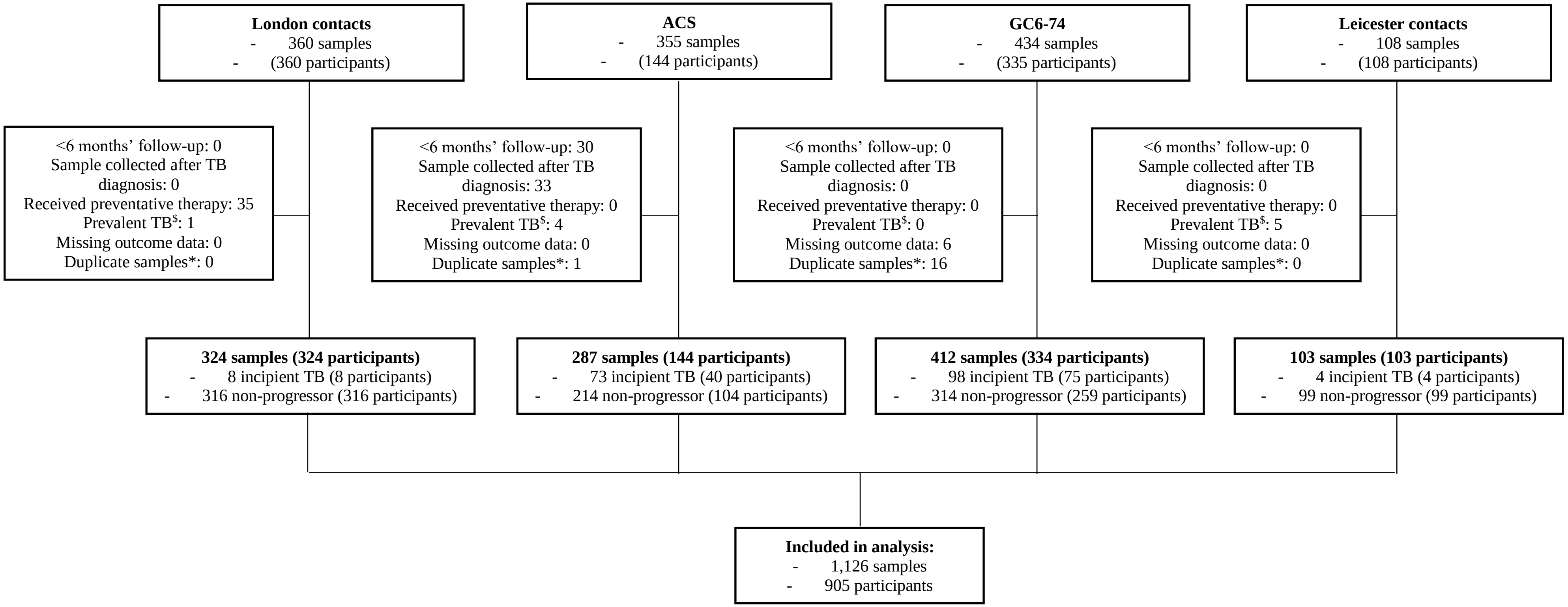

Supplementary Table 2. Baseline characteristics of participants in meta-analysis of concise whole blood transcriptional signatures for incipient tuberculosis (TB), stratified by study. ACS = adolescent cohort study; GC6-74 = Bill and Melinda Gates Foundation Grand Challenges 6-74 TB contacts study; IGRA = interferon gamma release assay; IQR = interquartile range. Data shown as n(%) unless otherwise specified.

Category	Level	London contacts	Leicester contacts	GC6-74	ACS	All
**Participants**	*n*	324	103	334	144	905
**Age**	*Median (IQR)*	34.00 (26.00, 47.00)	35.00 (24.00, 45.50)	23.00 (19.00, 35.00)	16.00 (15.00, 17.00)	26.00 (18.00, 40.00)
**Gender**	*Female*	153 (47.2)	43 (41.7)	197 (59.0)	97 (67.4)	490 (54.1)
	Male	166 (51.2)	60 (58.3)	137 (41.0)	47 (32.6)	410 (45.3)
	Missing	5 (1.5)	0 (0.0)	0 (0.0)	0 (0.0)	5 (0.6)
**Ethnicity**	*White*	75 (23.1)	6 (5.8)	0 (0.0)	0 (0.0)	81 (9.0)
	Black African or Caribbean	66 (20.4)	10 (9.7)	0 (0.0)	12 (8.3)	88 (9.7)
	South Asian	112 (34.6)	86 (83.5)	0 (0.0)	0 (0.0)	198 (21.9)
	Mixed	0 (0.0)	0 (0.0)	0 (0.0)	132 (91.7)	132 (14.6)
	Other	61 (18.8)	1 (1.0)	0 (0.0)	0 (0.0)	62 (6.9)
	Missing	10 (3.1)	0 (0.0)	334 (100.0)	0 (0.0)	344 (38.0)
**IGRA**	*Negative*	219 (67.6)	50 (48.5)	0 (0.0)	3 (2.1)	272 (30.1)
	Positive	105 (32.4)	53 (51.5)	0 (0.0)	36 (25.0)	194 (21.4)
	Missing	0 (0.0)	0 (0.0)	334 (100.0)	105 (72.9)	439 (48.5)
**Country**	*Ethiopia*	0 (0.0)	0 (0.0)	36 (10.8)	0 (0.0)	36 (4.0)
	South Africa	0 (0.0)	0 (0.0)	180 (53.9)	144 (100.0)	324 (35.8)
	The Gambia	0 (0.0)	0 (0.0)	118 (35.3)	0 (0.0)	118 (13.0)
	UK	324 (100.0)	103 (100.0)	0 (0.0)	0 (0.0)	427 (47.2)
**Outcome**	*Non-progressor*	316 (97.5)	99 (96.1)	259 (77.5)	104 (72.2)	778 (86.0)
	Incipient TB	8 (2.5)	4 (3.9)	75 (22.5)	40 (27.8)	127 (14.0)
**Months from recruitment to TB**	*Median (IQR)*	8.63 (6.39, 11.08)	1.92 (1.00, 3.16)	10.50 (5.50, 17.50)	14.37 (8.80, 18.62)	10.27 (5.50, 17.50)
**Microbiological confirmation**	*No*	5 (62.5)	0 (0.0)	5 (6.7)	0 (0.0)	10 (7.9)
	Yes	3 (37.5)	4 (100.0)	70 (93.3)	40 (100.0)	117 (92.1)
**Pulmonary**	*No*	7 (87.5)	1 (25.0)	0 (0.0)	0 (0.0)	8 (6.3)
	Yes	1 (12.5)	3 (75.0)	75 (100.0)	40 (100.0)	119 (93.7)
**Samples**	*n*	324	103	412	287	1126
	Non-progressor	316 (97.5)	99 (96.1)	314 (76.2)	214 (74.6)	943 (83.7)
	Incipient TB	8 (2.5)	4 (3.9)	98 (23.8)	73 (25.4)	183 (16.3)
**Months from sample to TB**	*Median (IQR)*	8.63 (6.39, 11.08)	1.92 (1.00, 3.16)	7.50 (5.50, 15.50)	9.33 (6.57, 14.97)	8.50 (5.50, 15.10)
	<3	1 (12.5)	3 (75.0)	6 (6.1)	11 (15.1)	21 (11.5)
	3 to 6	1 (12.5)	1 (25.0)	35 (35.7)	3 (4.1)	40 (21.9)
	6 to 12	5 (62.5)	0 (0.0)	23 (23.5)	28 (38.4)	56 (30.6)
	>12	1 (12.5)	0 (0.0)	34 (34.7)	31 (42.5)	66 (36.1)

**Samples per patient**	*1*	324 (100.0)	103 (100.0)	262 (78.4)	78 (54.2)	767 (84.8)
	2	0 (0.0)	0 (0.0)	66 (19.8)	22 (15.3)	88 (9.7)
	3	0 (0.0)	0 (0.0)	6 (1.8)	11 (7.6)	17 (1.9)
	4	0 (0.0)	0 (0.0)	0 (0.0)	33 (22.9)	33 (3.6)

Supplementary Figure 3. Principal component analysis (PCA) of RNAseq before batch correction showing (a) the entire transcriptome; (b) selected genes comprising only the candidate signatures included in the analysis; and (c) invariant genes, stratified by source study. Panel (d) shows PCA following batch correction. ACS = adolescent cohort study; GC6-74 = Bill and Melinda Gates Foundation Grand Challenges 6-74 TB contacts study.

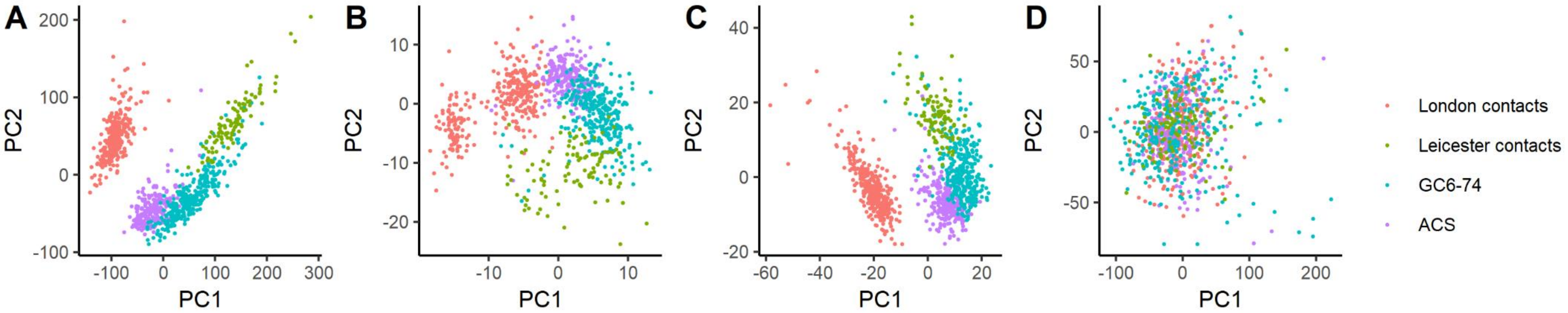

Supplementary Table 3. Validation of reconstructed signature models against the authors' original descriptions by comparing receiver operating characteristic areas under the curves (AUCs) in common datasets. No validation was possible for the Huang11 model as no AUC reported by authors in their original training / test set

Signature	Original AUC	Our AUC	Common dataset
*Zak16**	0.69	0.71	Zak test (GSE79362)
Suliman4[$]	0.67	0.66	Suliman test (GSE94438)
*Walter45**	0.98	0.98	Walter test (GSE73408)
Maertzdorf4[%]	0.98	1.00	Maertzdorf training (GSE74092)

**Support vector machine models including individual genes with linear kernels using the R kernlab package.*
[$]*Also reconstructed as a support vector machine model using gene pairs. Since performance was marginally better and closer to the authors' AUC in their own test set using ((GAS6 + SEPT4) - (CD1C + BLK); AUC=0.66), compared to the gene pairs SVM (AUC=0.65), the simple formula approach was used.*
[%]*Random forest model created using randomForest package in R.*

Supplementary Figure 4. Density plots of signature expression (a) before and (b) after batch correction, stratified by source study. ACS = adolescent cohort study; GC6-74 = Bill and Melinda Gates Foundation Grand Challenges 6-74 TB contacts study.

A

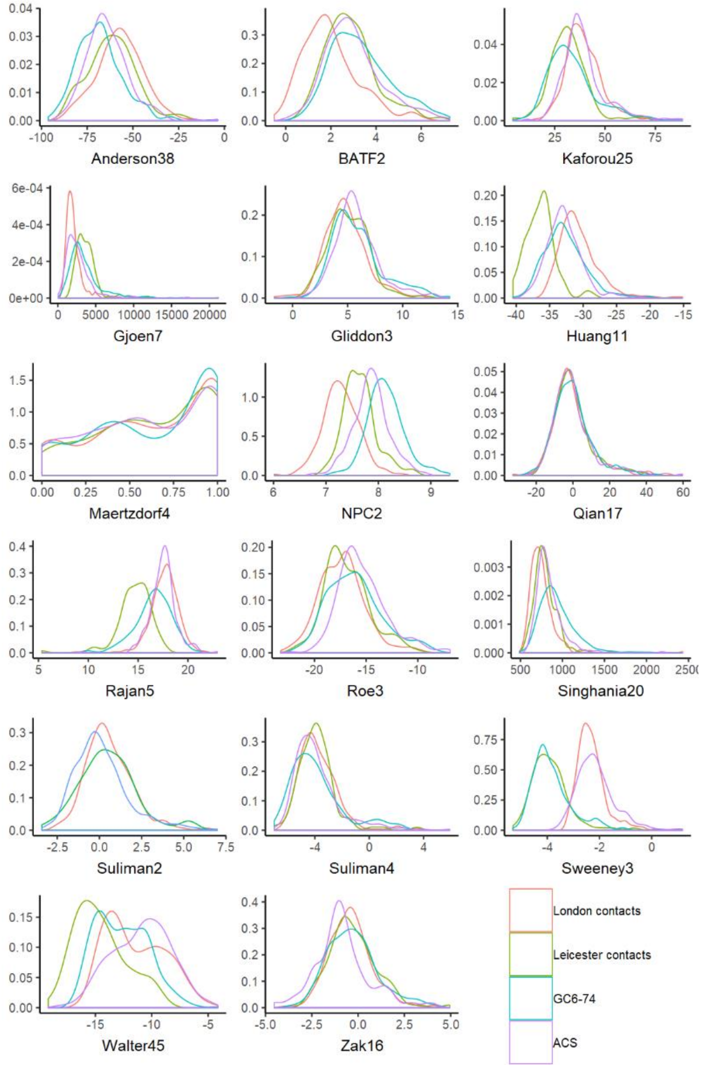

B

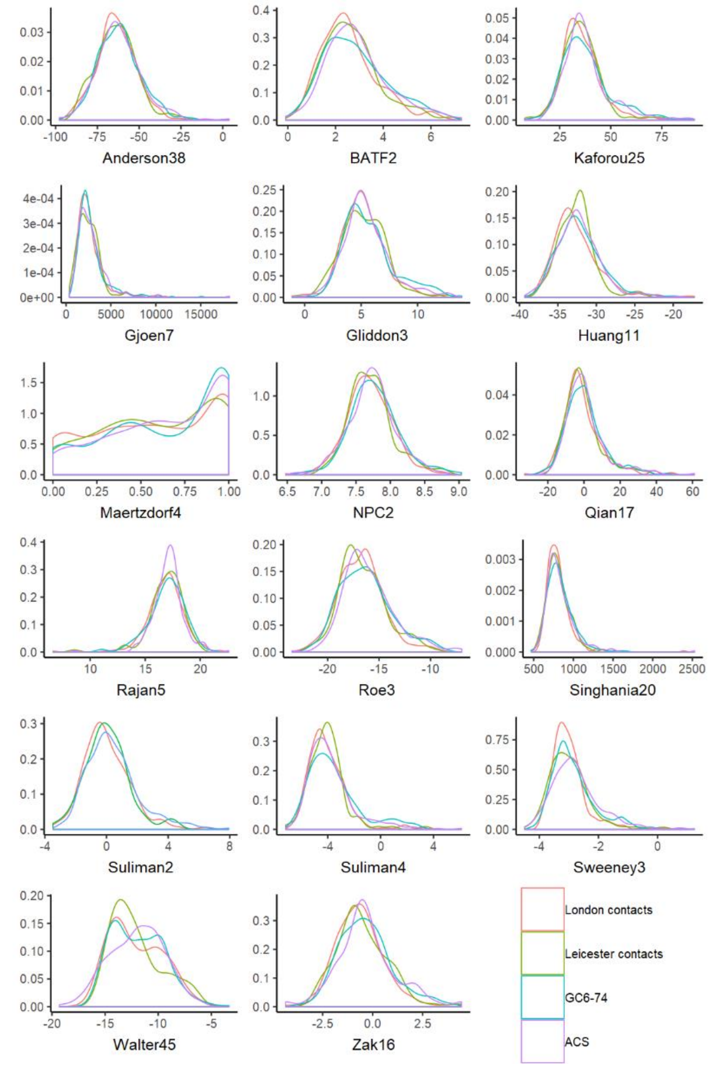

Supplementary Table 4. Tables showing receiver operating characteristic areas under the curve (95% confidence intervals) for 17 transcriptional signatures for identification of incipient TB over a two-year period, stratified by (a) study, and (b) study and time interval to disease. ACS = adolescent cohort study; GC6-74 = Bill and Melinda Gates Foundation Grand Challenges 6-74 TB contacts study.

(a)

Signature	ACS	GC6-74	London contacts	Leicester contacts
**Anderson38**	0.71 (0.63 - 0.79)	0.63 (0.56 - 0.7)	0.72 (0.56 - 0.88)	0.66 (0.41 - 0.9)
**BATF2**	0.81 (0.74 - 0.88)	0.68 (0.61 - 0.76)	0.81 (0.61 - 1)	0.71 (0.33 - 1)
**Gjoen7**	0.72 (0.64 - 0.8)	0.64 (0.57 - 0.71)	0.83 (0.71 - 0.94)	0.66 (0.28 - 1)
**Gliddon3**	0.72 (0.64 - 0.8)	0.74 (0.67 - 0.8)	0.84 (0.62 - 1)	0.66 (0.21 - 1)
**Huang11**	0.69 (0.61 - 0.77)	0.67 (0.6 - 0.74)	0.66 (0.46 - 0.85)	0.62 (0.25 - 0.98)
**Kaforou25**	0.79 (0.72 - 0.86)	0.7 (0.63 - 0.77)	0.84 (0.64 - 1)	0.66 (0.22 - 1)
**Maertzdorf4**	0.75 (0.68 - 0.83)	0.63 (0.56 - 0.7)	0.81 (0.67 - 0.95)	0.66 (0.23 - 1)
**NPC2**	0.64 (0.55 - 0.72)	0.69 (0.62 - 0.75)	0.84 (0.71 - 0.97)	0.8 (0.64 - 0.96)
**Qian17**	0.7 (0.62 - 0.78)	0.61 (0.54 - 0.68)	0.77 (0.61 - 0.94)	0.71 (0.44 - 0.97)
**Rajan5**	0.7 (0.62 - 0.78)	0.54 (0.47 - 0.61)	0.45 (0.17 - 0.72)	0.53 (0.32 - 0.74)
**Roe3**	0.83 (0.76 - 0.89)	0.64 (0.56 - 0.71)	0.79 (0.6 - 0.99)	0.74 (0.33 - 1)
**Singhania20**	0.69 (0.61 - 0.77)	0.66 (0.6 - 0.73)	0.73 (0.61 - 0.85)	0.45 (0.12 - 0.78)
**Suliman2**	0.8 (0.74 - 0.87)	NA	0.8 (0.63 - 0.98)	0.62 (0.21 - 1)
**Suliman4**	0.75 (0.67 - 0.83)	0.63 (0.52 - 0.74)	0.85 (0.72 - 0.98)	0.7 (0.28 - 1)
**Sweeney3**	0.78 (0.71 - 0.85)	0.69 (0.62 - 0.76)	0.75 (0.55 - 0.95)	0.65 (0.22 - 1)
**Walter45**	0.59 (0.5 - 0.68)	0.54 (0.47 - 0.62)	0.49 (0.27 - 0.71)	0.53 (0.35 - 0.71)
**Zak16**	0.69 (0.5 - 0.88)	0.67 (0.6 - 0.74)	0.79 (0.59 - 0.99)	0.76 (0.43 - 1)

(b)

Months to TB	12 to 24		6 to 12		3 to 6		0 to 3	
**Study**	**GC6-74**	**ACS**	**GC6-74**	**ACS**	**GC6-74**	**ACS**	**GC6-74**	**ACS**
**Anderson38**	0.58 (0.47 - 0.69)	0.71 (0.59 - 0.82)	0.72 (0.6 - 0.85)	0.65 (0.54 - 0.77)	0.64 (0.54 - 0.75)	0.59 (0.26 - 0.92)	0.61 (0.35 - 0.88)	0.8 (0.68 - 0.93)
**BATF2**	0.62 (0.5 - 0.74)	0.72 (0.61 - 0.83)	0.73 (0.6 - 0.86)	0.8 (0.7 - 0.9)	0.72 (0.62 - 0.83)	0.82 (0.57 - 1)	0.79 (0.55 - 1)	0.9 (0.82 - 0.98)
**Gjoen7**	0.58 (0.47 - 0.7)	0.69 (0.58 - 0.8)	0.7 (0.57 - 0.83)	0.68 (0.56 - 0.81)	0.68 (0.58 - 0.77)	0.76 (0.6 - 0.93)	0.68 (0.37 - 1)	0.74 (0.55 - 0.94)
**Gliddon3**	0.64 (0.53 - 0.76)	0.6 (0.49 - 0.7)	0.8 (0.68 - 0.92)	0.73 (0.64 - 0.82)	0.76 (0.67 - 0.85)	0.76 (0.45 - 1)	0.81 (0.57 - 1)	0.85 (0.7 - 1)
**Huang11**	0.56 (0.43 - 0.68)	0.59 (0.47 - 0.7)	0.78 (0.68 - 0.88)	0.72 (0.63 - 0.81)	0.6 (0.49 - 0.71)	0.69 (0.25 - 1)	0.92 (0.85 - 0.99)	0.7 (0.53 - 0.87)
**Kaforou25**	0.61 (0.49 - 0.73)	0.69 (0.58 - 0.79)	0.78 (0.65 - 0.91)	0.79 (0.71 - 0.87)	0.73 (0.64 - 0.83)	0.81 (0.57 - 1)	0.84 (0.63 - 1)	0.89 (0.78 - 1)
**Maertzdorf4**	0.55 (0.43 - 0.67)	0.66 (0.56 - 0.77)	0.76 (0.64 - 0.88)	0.74 (0.62 - 0.86)	0.65 (0.54 - 0.75)	0.86 (0.73 - 0.98)	0.71 (0.4 - 1)	0.85 (0.73 - 0.97)
**NPC2**	0.61 (0.5 - 0.71)	0.59 (0.47 - 0.72)	0.68 (0.53 - 0.83)	0.63 (0.52 - 0.75)	0.73 (0.64 - 0.81)	0.67 (0.32 - 1)	0.9 (0.8 - 1)	0.69 (0.5 - 0.89)
**Qian17**	0.55 (0.43 - 0.67)	0.57 (0.45 - 0.7)	0.7 (0.58 - 0.82)	0.74 (0.64 - 0.85)	0.62 (0.51 - 0.73)	0.76 (0.39 - 1)	0.78 (0.58 - 0.99)	0.78 (0.64 - 0.91)
**Rajan5**	0.55 (0.44 - 0.66)	0.6 (0.49 - 0.71)	0.48 (0.34 - 0.61)	0.72 (0.62 - 0.82)	0.52 (0.41 - 0.62)	0.68 (0.31 - 1)	0.64 (0.35 - 0.93)	0.77 (0.61 - 0.93)
**Roe3**	0.55 (0.43 - 0.67)	0.75 (0.65 - 0.84)	0.7 (0.56 - 0.84)	0.83 (0.75 - 0.91)	0.71 (0.6 - 0.81)	0.82 (0.53 - 1)	0.77 (0.51 - 1)	0.91 (0.85 - 0.98)
**Singhania20**	0.64 (0.53 - 0.75)	0.66 (0.53 - 0.78)	0.63 (0.51 - 0.76)	0.65 (0.53 - 0.77)	0.7 (0.62 - 0.78)	0.65 (0.44 - 0.86)	0.65 (0.37 - 0.93)	0.81 (0.65 - 0.98)
**Suliman4**	0.61 (0.42 - 0.81)	0.68 (0.56 - 0.8)	0.66 (0.47 - 0.84)	0.7 (0.58 - 0.82)	0.75 (0.56 - 0.93)	0.83 (0.58 - 1)	0.81 (0.64 - 0.97)	0.91 (0.84 - 0.97)
**Sweeney3**	0.59 (0.48 - 0.71)	0.7 (0.6 - 0.8)	0.78 (0.66 - 0.89)	0.73 (0.62 - 0.83)	0.69 (0.58 - 0.8)	0.81 (0.5 - 1)	0.91 (0.77 - 1)	0.9 (0.79 - 1)
**Walter45**	0.54 (0.43 - 0.65)	0.45 (0.33 - 0.57)	0.53 (0.4 - 0.65)	0.57 (0.44 - 0.69)	0.69 (0.58 - 0.79)	0.55 (0.31 - 0.79)	0.53 (0.19 - 0.87)	0.47 (0.3 - 0.64)
**Zak16**	0.58 (0.46 - 0.69)	0.42 (0.19 - 0.65)	0.71 (0.57 - 0.85)	0.67 (0.36 - 0.99)	NA	NA	0.76 (0.47 - 1)	0.94 (0.86 - 1)

Supplementary Table 5. Receiver operating characteristic areas under the curve (95% confidence intervals) showing diagnostic accuracy of candidate transcriptional signatures for incipient TB, stratified by time interval to disease, among pooled dataset of four contributing studies. P values represent paired comparisons against the best performing signature available for all participants (BATF2) over two years, using DeLong tests. Number of samples included for each signature, at each time point, indicated in Supplementary Table 6.

Signature	0 to 24	p	0 to 12	0 to 6	0 to 3
**Suliman2**	0.77 (0.71 - 0.82)	0.356	0.82 (0.76 - 0.88)	0.85 (0.74 - 0.95)	0.91 (0.86 - 0.96)
**BATF2**	0.74 (0.69 - 0.78)	1.000	0.77 (0.72 - 0.82)	0.78 (0.7 - 0.85)	0.87 (0.79 - 0.95)
**Kaforou25**	0.73 (0.69 - 0.78)	0.852	0.78 (0.73 - 0.83)	0.79 (0.72 - 0.86)	0.88 (0.8 - 0.97)
**Gliddon3**	0.73 (0.68 - 0.77)	0.576	0.77 (0.72 - 0.82)	0.78 (0.71 - 0.85)	0.85 (0.74 - 0.96)
**Sweeney3**	0.72 (0.68 - 0.77)	0.438	0.77 (0.71 - 0.82)	0.77 (0.69 - 0.84)	0.91 (0.84 - 0.97)
**Roe3**	0.72 (0.67 - 0.77)	0.109	0.77 (0.71 - 0.82)	0.77 (0.7 - 0.84)	0.88 (0.79 - 0.97)
**Suliman4**	0.7 (0.64 - 0.76)	0.256	0.73 (0.66 - 0.8)	0.78 (0.68 - 0.89)	0.82 (0.69 - 0.94)
**Zak16**	0.7 (0.64 - 0.76)	0.939	0.76 (0.69 - 0.82)	0.79 (0.71 - 0.86)	0.86 (0.71 - 1)
**NPC2**	0.68 (0.64 - 0.73)	0.012	0.71 (0.66 - 0.77)	0.75 (0.69 - 0.82)	0.78 (0.66 - 0.9)
**Maertzdorf4**	0.68 (0.63 - 0.73)	0.001	0.73 (0.68 - 0.78)	0.71 (0.64 - 0.79)	0.8 (0.69 - 0.91)
**Gjoen7**	0.67 (0.63 - 0.72)	0.001	0.69 (0.64 - 0.75)	0.7 (0.62 - 0.77)	0.75 (0.61 - 0.88)
**Singhania20**	0.67 (0.62 - 0.72)	0.006	0.68 (0.62 - 0.73)	0.72 (0.65 - 0.78)	0.74 (0.6 - 0.87)
**Huang11**	0.66 (0.61 - 0.71)	0.007	0.7 (0.65 - 0.75)	0.67 (0.6 - 0.75)	0.75 (0.63 - 0.86)
**Qian17**	0.66 (0.61 - 0.71)	<0.0001	0.71 (0.66 - 0.76)	0.69 (0.62 - 0.77)	0.79 (0.7 - 0.88)
**Anderson38**	0.65 (0.61 - 0.7)	0.002	0.68 (0.62 - 0.73)	0.68 (0.6 - 0.75)	0.74 (0.63 - 0.85)
**Rajan5**	0.55 (0.5 - 0.6)	<0.0001	0.59 (0.53 - 0.65)	0.57 (0.49 - 0.66)	0.68 (0.56 - 0.81)
**Walter45**	0.55 (0.5 - 0.6)	<0.0001	0.58 (0.52 - 0.64)	0.62 (0.54 - 0.69)	0.47 (0.35 - 0.6)

Supplementary Figure 5. Density plots of signature expression of eight best performing transcriptional signatures for incipient tuberculosis, among control population with negative interferon-gamma release assay tests. Plots demonstrate approximately Normal distributions.

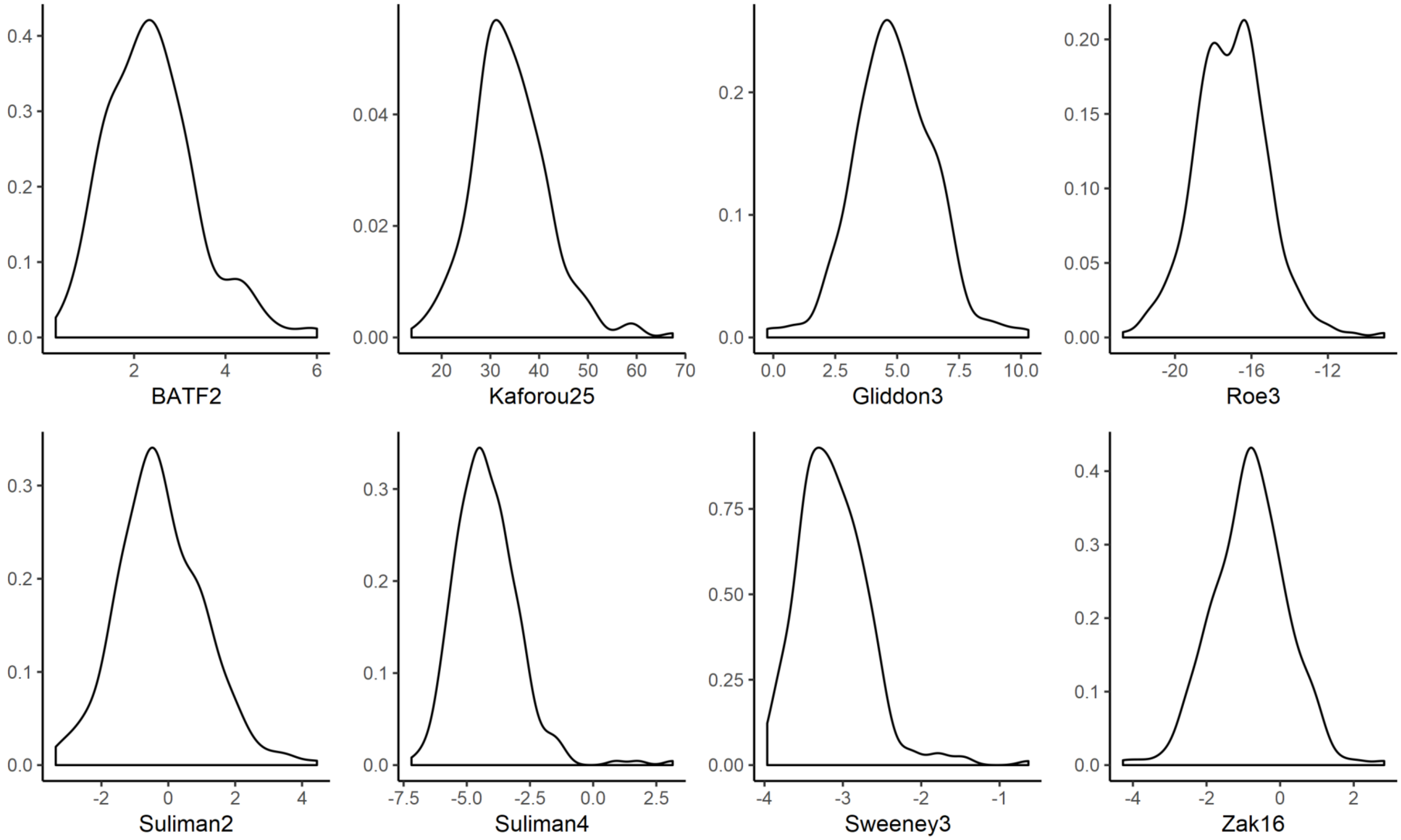

Supplementary Figure 6. Relationships between 17 candidate transcriptional signatures for incipient tuberculosis (TB) included in meta-analysis displayed as: (a) Spearman rank correlation matrix heatmap; (b) Jaccard similarity index heatmap showing overlapping constituent genes; and (c) Jaccard index *vs*. Spearman correlation coefficient for pairwise signature comparisons.

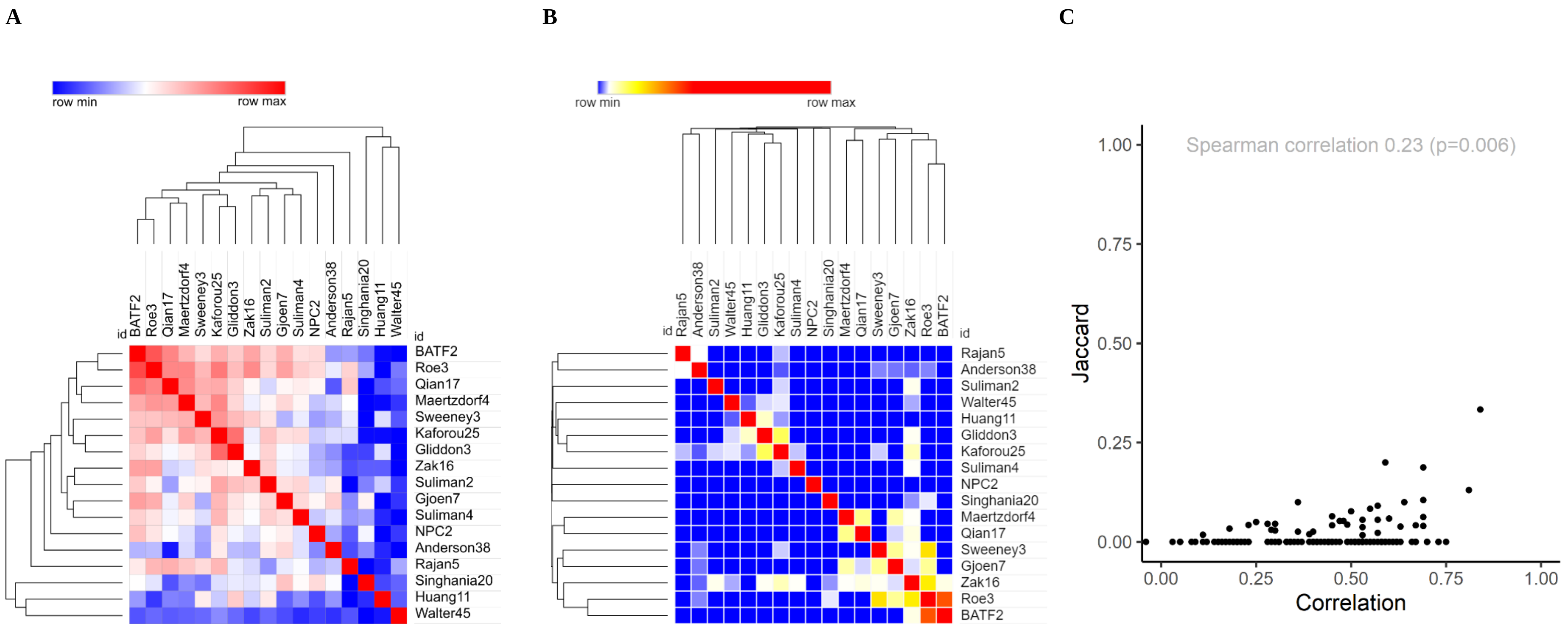

Supplementary Figure 7. Scatterplots showing expression of eight best performing transcriptional signatures among participants progressing to TB by days from sampling to disease. Horizontal dashed line indicates cut-off defined by two standard deviations above the mean of control population. Vertical grey dashed line indicates the initial 90 day interval after sampling.

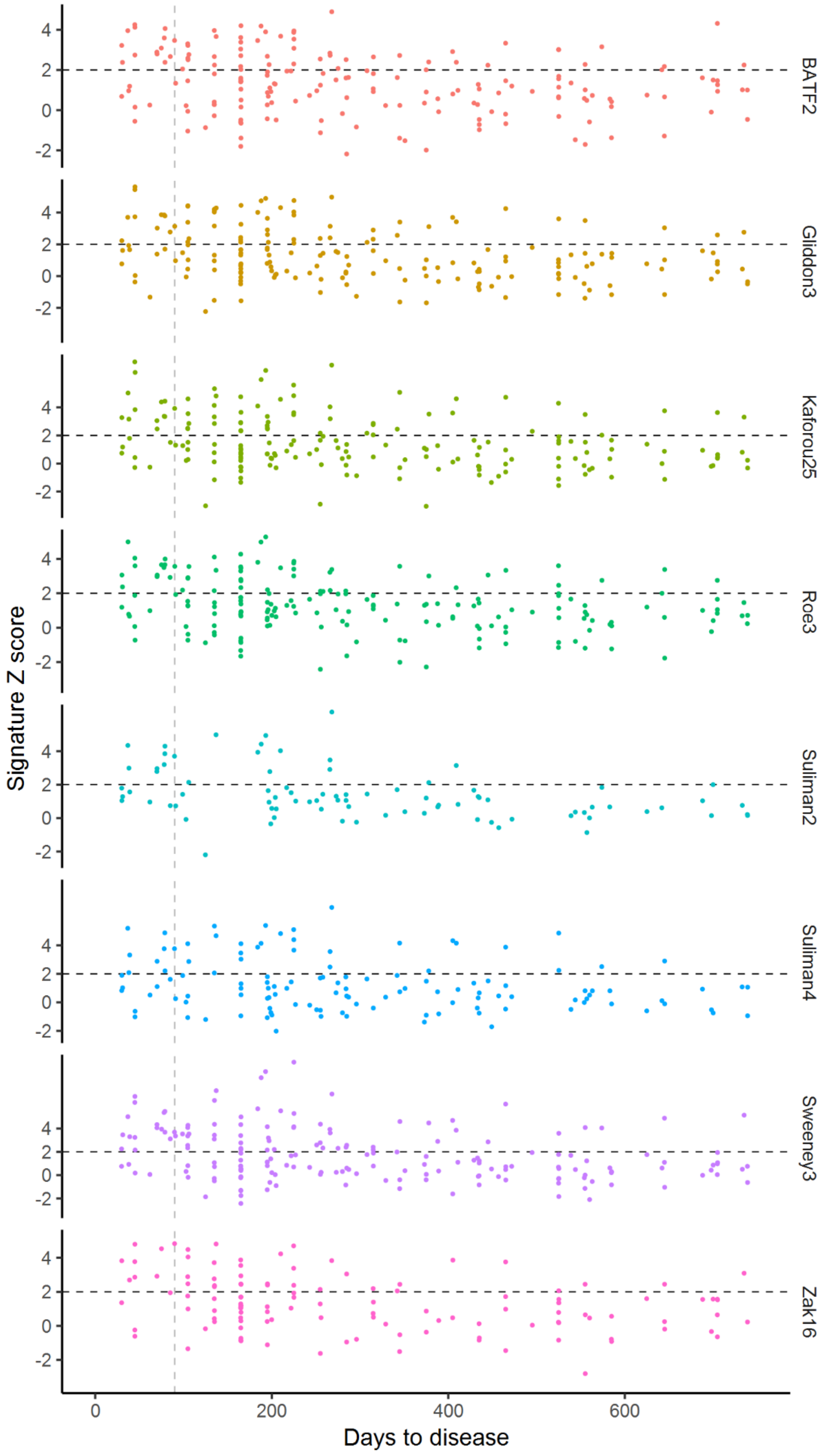

Supplementary Table 6. Number of incipient tuberculosis and non-progressor samples used in analysis in (a) primary analysis; (b) sensitivity analysis including only TB cases with microbiological confirmation; (c) sensitivity analysis including only one sample per participant; (d) sensitivity analysis with mutually exclusive time intervals to TB. Data presented as n (% of all available samples per time interval). 'All other signatures' includes Anderson38, BATF2, Gjoen7, Gliddon3, Huang11, Kaforou25, Maertzdorf4, NPC2, Qian17, Rajan5, Roe3, Singhania20, Sweeney3, and Walter45.

Timeframe	Signature	0 to 24 months		0 to 12 months		0 to 6 months		0 to 3 months	
		Non-progressor	*Incipient TB*	*Non-progressor*	*Incipient TB*	*Non-progressor*	*Incipient TB*	*Non-progressor*	*Incipient TB*
**(a) Primary analysis**	*Zak16*	489 (87.5)	119 (66.9)	723 (85.1)	80 (68.4)	777 (82.4)	49 (80.3)	777 (82.4)	12 (57.1)
	Suliman4	444 (79.4)	122 (68.5)	706 (83.1)	81 (69.2)	771 (81.8)	34 (55.7)	771 (81.8)	17 (81.0)
	Suliman2	351 (62.8)	81 (45.5)	584 (68.7)	53 (45.3)	629 (66.7)	20 (32.8)	629 (66.7)	15 (71.4)
	All other signatures	559 (100.0)	178 (100.0)	850 (100.0)	117 (100.0)	943 (100.0)	61 (100.0)	943 (100.0)	21 (100.0)
**(b) Microbiological confirmation**	*Zak16*	489 (87.5)	106 (64.2)	723 (85.1)	70 (65.4)	777 (82.4)	44 (78.6)	777 (82.4)	11 (55.0)
	Suliman4	444 (79.4)	111 (67.3)	706 (83.1)	73 (68.2)	771 (81.8)	30 (53.6)	771 (81.8)	16 (80.0)
	Suliman2	351 (62.8)	76 (46.1)	584 (68.7)	49 (45.8)	629 (66.7)	18 (32.1)	629 (66.7)	14 (70.0)
	All other signatures	559 (100.0)	165 (100.0)	850 (100.0)	107 (100.0)	943 (100.0)	56 (100.0)	943 (100.0)	20 (100.0)
**(c) One sample per participant**	*Zak16*	463 (91.3)	94 (74.6)	676 (91.8)	64 (73.6)	704 (90.5)	41 (85.4)	704 (90.5)	10 (66.7)
	Suliman4	406 (80.1)	81 (64.3)	609 (82.7)	58 (66.7)	638 (82.0)	24 (50.0)	638 (82.0)	11 (73.3)
	Suliman2	319 (62.9)	51 (40.5)	504 (68.5)	37 (42.5)	519 (66.7)	14 (29.2)	519 (66.7)	10 (66.7)
	All other signatures	507 (100.0)	126 (100.0)	736 (100.0)	87 (100.0)	778 (100.0)	48 (100.0)	778 (100.0)	15 (100.0)
**(d) Mutually exclusive time periods**		**12 to 24 months**		**6 to 12 months**		**3 to 6 months**		**0 to 3 months**	
	Zak16	489 (87.5)	39 (63.9)	723 (85.1)	31 (55.4)	777 (82.4)	37 (92.5)	777 (82.4)	12 (57.1)
	Suliman4	444 (79.4)	41 (67.2)	706 (83.1)	47 (83.9)	771 (81.8)	17 (42.5)	771 (81.8)	17 (81.0)
	Suliman2	351 (62.8)	28 (45.9)	584 (68.7)	33 (58.9)	629 (66.7)	5 (12.5)	629 (66.7)	15 (71.4)
	All other signatures	559 (100.0)	61 (100.0)	850 (100.0)	56 (100.0)	943 (100.0)	40 (100.0)	943 (100.0)	21 (100.0)

Supplementary Table 7. Diagnostic accuracy of eight best performing transcriptional signatures for incipient tuberculosis (TB), stratified by months to disease, using pre-specified Z2 cut-offs based on the 97.7th percentile of the control population . Positive and negative predictive values (PPVs/NPVs) shown assuming 2% pre-test probability. Data presented as estimate (95% confidence interval). Data presented graphically in Figure 4. Number of samples included for each signature, at each time point, indicated in Supplementary Table 6.

**Timeframe**	**0 to 24**			
**Signature**	**Sensitivity**	**Specificity**	**PPV**	**NPV**
**BATF2**	0.35 (0.29 - 0.43)	0.93 (0.91 - 0.95)	0.074 (0.049 - 0.108)	0.986 (0.984 - 0.988)
**Gliddon3**	0.31 (0.25 - 0.39)	0.95 (0.93 - 0.97)	0.085 (0.054 - 0.128)	0.986 (0.984 - 0.987)
**Kaforou25**	0.35 (0.28 - 0.42)	0.95 (0.93 - 0.97)	0.094 (0.06 - 0.138)	0.986 (0.985 - 0.988)
**Roe3**	0.3 (0.24 - 0.37)	0.94 (0.92 - 0.96)	0.07 (0.044 - 0.105)	0.985 (0.983 - 0.987)
**Suliman2**	0.25 (0.17 - 0.35)	0.95 (0.92 - 0.97)	0.068 (0.034 - 0.123)	0.984 (0.982 - 0.987)
**Suliman4**	0.29 (0.21 - 0.37)	0.95 (0.93 - 0.97)	0.08 (0.046 - 0.129)	0.985 (0.983 - 0.987)
**Sweeney3**	0.4 (0.33 - 0.47)	0.92 (0.9 - 0.94)	0.077 (0.052 - 0.11)	0.987 (0.985 - 0.989)
**Zak16**	0.33 (0.25 - 0.42)	0.94 (0.91 - 0.95)	0.074 (0.045 - 0.115)	0.986 (0.983 - 0.988)
**Timeframe**	**0 to 12**			
**BATF2**	0.43 (0.34 - 0.52)	0.92 (0.9 - 0.94)	0.083 (0.056 - 0.116)	0.988 (0.985 - 0.99)
**Gliddon3**	0.41 (0.33 - 0.5)	0.94 (0.92 - 0.95)	0.093 (0.062 - 0.132)	0.987 (0.985 - 0.989)
**Kaforou25**	0.44 (0.36 - 0.53)	0.94 (0.92 - 0.96)	0.103 (0.07 - 0.143)	0.988 (0.986 - 0.99)
**Roe3**	0.37 (0.29 - 0.46)	0.94 (0.92 - 0.95)	0.085 (0.056 - 0.123)	0.986 (0.984 - 0.989)
**Suliman2**	0.34 (0.23 - 0.47)	0.95 (0.92 - 0.96)	0.084 (0.046 - 0.14)	0.986 (0.983 - 0.989)
**Suliman4**	0.33 (0.24 - 0.44)	0.95 (0.93 - 0.96)	0.089 (0.052 - 0.138)	0.986 (0.984 - 0.988)
**Sweeney3**	0.52 (0.43 - 0.61)	0.92 (0.89 - 0.93)	0.092 (0.065 - 0.124)	0.989 (0.987 - 0.992)
**Zak16**	0.42 (0.32 - 0.53)	0.94 (0.92 - 0.95)	0.096 (0.061 - 0.142)	0.988 (0.985 - 0.99)
**Timeframe**	**0 to 6**			
**BATF2**	0.51 (0.39 - 0.63)	0.93 (0.91 - 0.94)	0.099 (0.065 - 0.139)	0.989 (0.986 - 0.992)
**Gliddon3**	0.43 (0.31 - 0.55)	0.94 (0.92 - 0.95)	0.093 (0.058 - 0.137)	0.988 (0.985 - 0.99)
**Kaforou25**	0.48 (0.36 - 0.6)	0.94 (0.92 - 0.95)	0.102 (0.066 - 0.147)	0.989 (0.986 - 0.991)
**Roe3**	0.44 (0.33 - 0.57)	0.94 (0.92 - 0.95)	0.098 (0.062 - 0.143)	0.988 (0.985 - 0.991)
**Suliman2**	0.5 (0.3 - 0.7)	0.94 (0.92 - 0.95)	0.11 (0.055 - 0.178)	0.989 (0.985 - 0.994)
**Suliman4**	0.47 (0.31 - 0.63)	0.95 (0.93 - 0.96)	0.117 (0.066 - 0.18)	0.989 (0.985 - 0.992)
**Sweeney3**	0.57 (0.45 - 0.69)	0.91 (0.89 - 0.93)	0.095 (0.065 - 0.129)	0.991 (0.988 - 0.993)
**Zak16**	0.45 (0.32 - 0.59)	0.94 (0.92 - 0.95)	0.102 (0.061 - 0.153)	0.988 (0.985 - 0.991)
**Timeframe**	**0 to 3**			
**BATF2**	0.67 (0.45 - 0.83)	0.93 (0.91 - 0.94)	0.126 (0.076 - 0.175)	0.993 (0.988 - 0.996)
**Gliddon3**	0.52 (0.32 - 0.72)	0.94 (0.92 - 0.95)	0.112 (0.06 - 0.171)	0.99 (0.985 - 0.994)
**Kaforou25**	0.62 (0.41 - 0.79)	0.94 (0.92 - 0.95)	0.129 (0.075 - 0.185)	0.992 (0.987 - 0.996)
**Roe3**	0.62 (0.41 - 0.79)	0.94 (0.92 - 0.95)	0.132 (0.077 - 0.189)	0.992 (0.987 - 0.996)
**Suliman2**	0.53 (0.3 - 0.75)	0.94 (0.92 - 0.95)	0.117 (0.056 - 0.188)	0.99 (0.985 - 0.995)
**Suliman4**	0.47 (0.26 - 0.69)	0.95 (0.93 - 0.96)	0.117 (0.056 - 0.193)	0.989 (0.984 - 0.993)
**Sweeney3**	0.81 (0.6 - 0.92)	0.91 (0.89 - 0.93)	0.129 (0.085 - 0.166)	0.996 (0.991 - 0.998)
**Zak16**	0.67 (0.39 - 0.86)	0.94 (0.92 - 0.95)	0.144 (0.074 - 0.21)	0.993 (0.987 - 0.997)

Supplementary Figure 8. Positive- and negative-predictive values (PPVs/NPVs), modelled across a range of pre-test probabilities for eight best performing transcriptional signatures for incipient tuberculosis (TB), stratified by months to disease, using pre-specified Z2 cut-offs based on the 97.7th percentile of the control population. Dashed line indicates 2% pre-test probability.

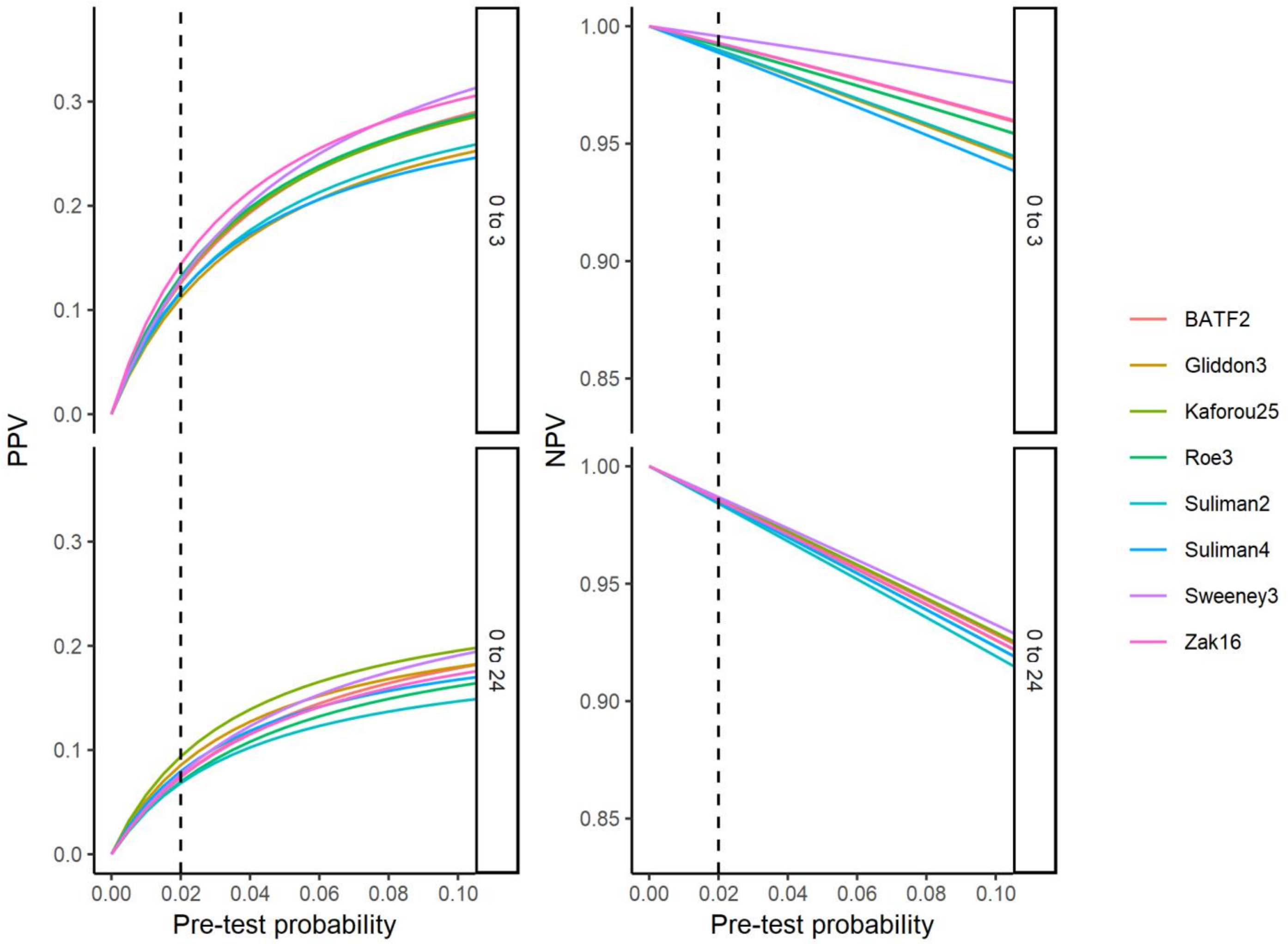

Supplementary Figure 9. Diagnostic accuracy of eight best performing transcriptional signatures for incipient tuberculosis (TB) shown in receiver operating characteristic space, stratified by months to disease. Grey shaded zones indicate 95% confidence intervals for each signature. Sensitivity analysis presented using biomarkers cut-offs defined by the maximal Youden indices for each time period, benchmarked against minimal (grey dashed box) and optimal (black dashed box) criteria from the WHO Target Product Profile for incipient TB biomarkers. Point estimates and 95% confidence intervals shown in Supplementary Table 8. Number of samples included for each signature, at each time point, indicated in Supplementary Table 6.

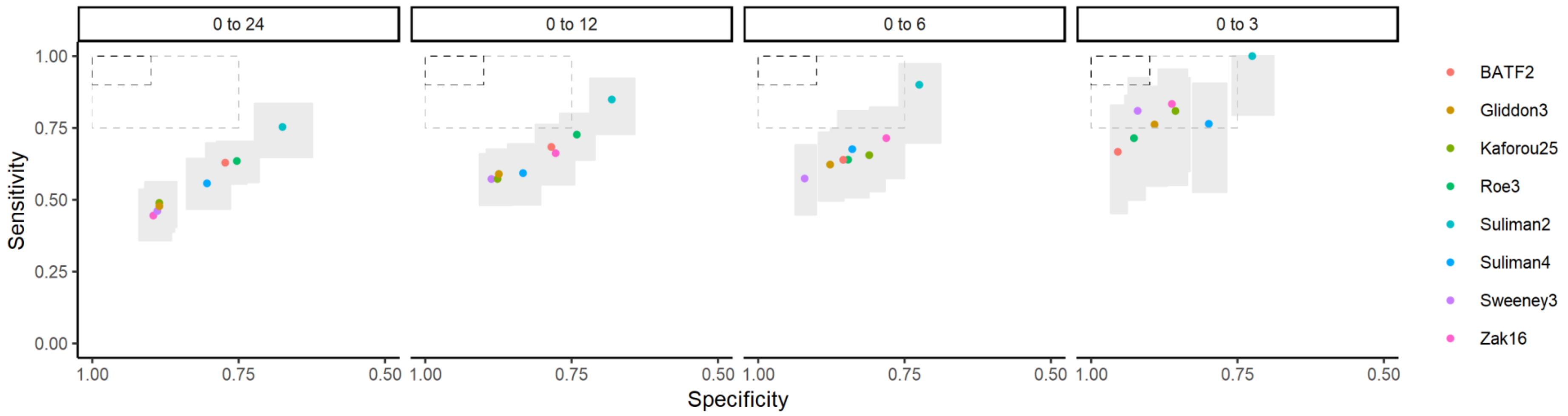

Supplementary Table 8. Diagnostic accuracy of eight best performing transcriptional signatures for incipient tuberculosis (TB), stratified by months to disease, using cut-offs defined by the maximal Youden index for each time interval. Positive and negative predictive values (PPVs/NPVs) shown assuming 2% pre-test probability. Data presented as estimate (95% confidence interval). Data presented graphically in Supplementary Figure 9. Number of samples included for each signature, at each time point, indicated in Supplementary Table 6.

**Timeframe**	**0 to 24**			
**Signature**	**Sensitivity**	**Specificity**	**PPV**	**NPV**
**BATF2**	0.63 (0.56 - 0.7)	0.77 (0.74 - 0.81)	0.049 (0.038 - 0.062)	0.99 (0.988 - 0.992)
**Gliddon3**	0.48 (0.41 - 0.55)	0.89 (0.86 - 0.91)	0.067 (0.048 - 0.092)	0.988 (0.986 - 0.99)
**Kaforou25**	0.49 (0.42 - 0.56)	0.89 (0.86 - 0.91)	0.069 (0.049 - 0.094)	0.988 (0.986 - 0.99)
**Roe3**	0.63 (0.56 - 0.7)	0.75 (0.72 - 0.79)	0.046 (0.036 - 0.058)	0.99 (0.988 - 0.992)
**Suliman2**	0.75 (0.65 - 0.83)	0.68 (0.62 - 0.72)	0.043 (0.032 - 0.054)	0.993 (0.989 - 0.995)
**Suliman4**	0.56 (0.47 - 0.64)	0.8 (0.76 - 0.84)	0.05 (0.036 - 0.067)	0.989 (0.986 - 0.991)
**Sweeney3**	0.46 (0.39 - 0.53)	0.89 (0.86 - 0.91)	0.067 (0.047 - 0.092)	0.988 (0.986 - 0.99)
**Zak16**	0.45 (0.36 - 0.53)	0.9 (0.87 - 0.92)	0.068 (0.045 - 0.098)	0.988 (0.985 - 0.99)
**Timeframe**	**0 to 12**			
**BATF2**	0.68 (0.59 - 0.76)	0.78 (0.76 - 0.81)	0.056 (0.044 - 0.069)	0.992 (0.989 - 0.994)
**Gliddon3**	0.59 (0.5 - 0.67)	0.87 (0.85 - 0.89)	0.076 (0.056 - 0.099)	0.991 (0.988 - 0.993)
**Kaforou25**	0.57 (0.48 - 0.66)	0.88 (0.85 - 0.9)	0.075 (0.055 - 0.098)	0.99 (0.988 - 0.992)
**Roe3**	0.73 (0.64 - 0.8)	0.74 (0.71 - 0.77)	0.05 (0.04 - 0.061)	0.993 (0.99 - 0.995)
**Suliman2**	0.85 (0.73 - 0.92)	0.68 (0.64 - 0.72)	0.049 (0.038 - 0.059)	0.996 (0.991 - 0.998)
**Suliman4**	0.59 (0.48 - 0.69)	0.83 (0.8 - 0.86)	0.061 (0.044 - 0.08)	0.99 (0.987 - 0.993)
**Sweeney3**	0.57 (0.48 - 0.66)	0.89 (0.86 - 0.91)	0.081 (0.059 - 0.106)	0.99 (0.988 - 0.992)
**Zak16**	0.66 (0.55 - 0.76)	0.78 (0.75 - 0.81)	0.053 (0.039 - 0.067)	0.991 (0.988 - 0.994)
**Timeframe**	**0 to 6**			
**BATF2**	0.64 (0.51 - 0.75)	0.85 (0.83 - 0.88)	0.073 (0.052 - 0.095)	0.991 (0.988 - 0.994)
**Gliddon3**	0.62 (0.5 - 0.73)	0.88 (0.85 - 0.9)	0.081 (0.058 - 0.108)	0.991 (0.988 - 0.994)
**Kaforou25**	0.66 (0.53 - 0.76)	0.81 (0.78 - 0.83)	0.06 (0.044 - 0.077)	0.991 (0.988 - 0.994)
**Roe3**	0.64 (0.51 - 0.75)	0.85 (0.82 - 0.87)	0.07 (0.05 - 0.091)	0.991 (0.988 - 0.994)
**Suliman2**	0.9 (0.7 - 0.97)	0.72 (0.69 - 0.76)	0.059 (0.041 - 0.07)	0.997 (0.991 - 0.999)
**Suliman4**	0.68 (0.51 - 0.81)	0.84 (0.81 - 0.86)	0.071 (0.047 - 0.095)	0.992 (0.988 - 0.995)
**Sweeney3**	0.57 (0.45 - 0.69)	0.92 (0.9 - 0.94)	0.105 (0.072 - 0.143)	0.991 (0.988 - 0.993)
**Zak16**	0.71 (0.58 - 0.82)	0.78 (0.75 - 0.81)	0.057 (0.042 - 0.073)	0.993 (0.989 - 0.996)
**Timeframe**	**0 to 3**			
**BATF2**	0.67 (0.45 - 0.83)	0.95 (0.94 - 0.97)	0.171 (0.102 - 0.237)	0.993 (0.988 - 0.996)
**Gliddon3**	0.76 (0.55 - 0.89)	0.89 (0.87 - 0.91)	0.108 (0.07 - 0.142)	0.995 (0.99 - 0.998)
**Kaforou25**	0.81 (0.6 - 0.92)	0.86 (0.83 - 0.88)	0.091 (0.061 - 0.116)	0.995 (0.99 - 0.998)
**Roe3**	0.71 (0.5 - 0.86)	0.93 (0.91 - 0.94)	0.135 (0.084 - 0.183)	0.994 (0.989 - 0.997)
**Suliman2**	1 (0.8 - 1)	0.72 (0.69 - 0.76)	0.065 (0.047 - 0.072)	1 (0.994 - 1)
**Suliman4**	0.76 (0.53 - 0.9)	0.8 (0.77 - 0.83)	0.066 (0.041 - 0.087)	0.994 (0.988 - 0.998)
**Sweeney3**	0.81 (0.6 - 0.92)	0.92 (0.9 - 0.94)	0.142 (0.093 - 0.183)	0.996 (0.991 - 0.998)
**Zak16**	0.83 (0.55 - 0.95)	0.86 (0.84 - 0.88)	0.097 (0.058 - 0.125)	0.996 (0.989 - 0.999)

Supplementary Figure 10. Receiver operating characteristic curves showing diagnostic accuracy of eight best performing transcriptional signatures for incipient tuberculosis (TB), stratified by months from sample collection to disease. Sensitivity analysis restricting inclusion of incipient TB cases to those with documented microbiological confirmation. Number of samples included for each signature, at each time point, indicated in Supplementary Table 6.

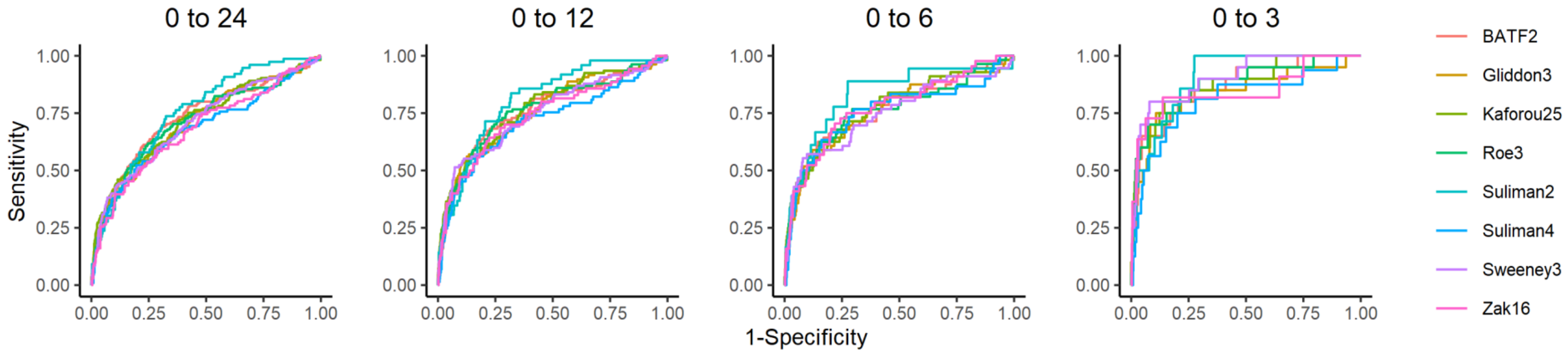

Supplementary Figure 11. Receiver operating characteristic curves showing diagnostic accuracy of eight best performing transcriptional signatures for incipient tuberculosis (TB), stratified by months from sample collection to disease. Sensitivity analysis including only one blood RNA sample per participant (by randomly sampling). Number of samples included for each signature, at each time point, indicated in Supplementary Table 6.

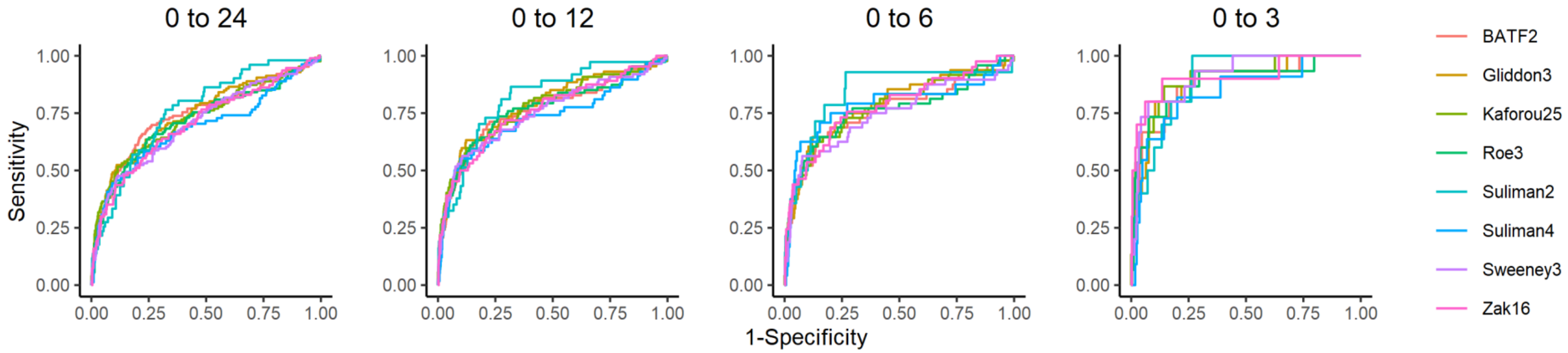

Supplementary Figure 12. Receiver operating characteristic curves showing diagnostic accuracy of eight best performing transcriptional signatures for incipient tuberculosis (TB), stratified by months from sample collection to disease. Sensitivity analysis using mutually exclusive time periods of 0-3, 3-6, 6-12 and 12-24 months. Number of samples included for each signature, at each time point, indicated in Supplementary Table 6. Area under the curve estimates and 95% confidence intervals are shown in Supplementary Table 9.

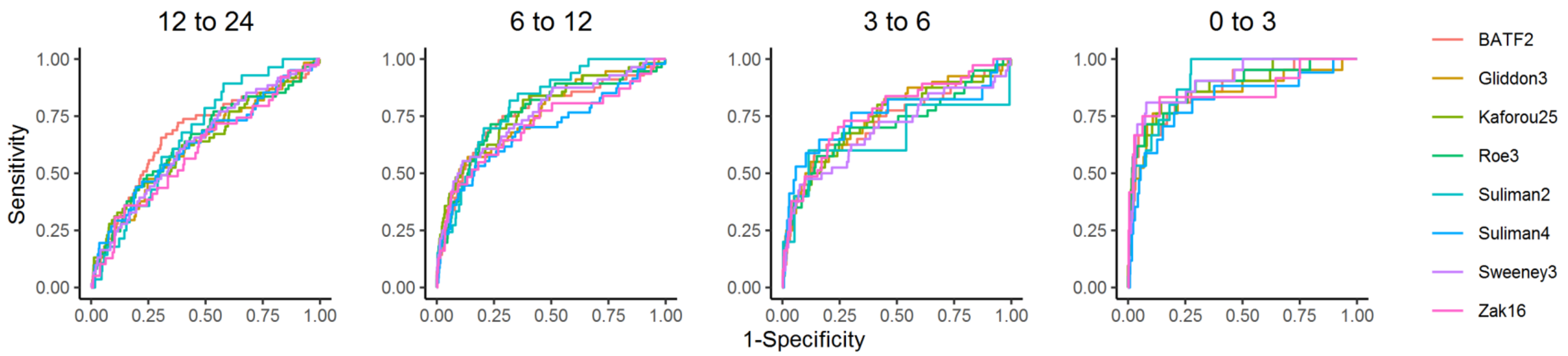

Supplementary Table 9. Receiver operating characteristic areas under the curve (95% confidence intervals) showing diagnostic accuracy of eight best performing transcriptional signatures for incipient tuberculosis (TB), stratified by months from sample collection to disease. Sensitivity analysis using mutually exclusive time periods of 0-3, 3-6, 6-12 and 12-24 months. Data presented graphically in Supplementary Figure 12. Number of samples included for each signature, at each time point, indicated in Supplementary Table 6.

**Signature**	**0 to 3**	**3 to 6**	**6 to 12**	**12 to 24**
**Suliman2**	0.91 (0.86 - 0.96)	0.66 (0.29 - 1)	0.8 (0.74 - 0.87)	0.67 (0.58 - 0.76)
**Sweeney3**	0.91 (0.84 - 0.97)	0.69 (0.59 - 0.8)	0.76 (0.69 - 0.83)	0.63 (0.56 - 0.71)
**Kaforou25**	0.88 (0.8 - 0.97)	0.74 (0.65 - 0.83)	0.78 (0.71 - 0.85)	0.63 (0.55 - 0.71)
**Roe3**	0.88 (0.79 - 0.97)	0.71 (0.61 - 0.81)	0.77 (0.69 - 0.84)	0.64 (0.56 - 0.72)
**BATF2**	0.87 (0.79 - 0.95)	0.73 (0.63 - 0.82)	0.76 (0.69 - 0.84)	0.67 (0.6 - 0.75)
**Zak16**	0.86 (0.71 - 1)	0.77 (0.68 - 0.85)	0.71 (0.6 - 0.82)	0.6 (0.5 - 0.7)
**Gliddon3**	0.85 (0.74 - 0.96)	0.75 (0.66 - 0.84)	0.76 (0.69 - 0.83)	0.62 (0.55 - 0.7)
**Suliman4**	0.82 (0.69 - 0.94)	0.75 (0.59 - 0.91)	0.69 (0.6 - 0.78)	0.63 (0.53 - 0.73)
**Maertzdorf4**	0.8 (0.69 - 0.91)	0.67 (0.57 - 0.76)	0.75 (0.67 - 0.82)	0.59 (0.51 - 0.67)
**Qian17**	0.79 (0.7 - 0.88)	0.64 (0.54 - 0.74)	0.73 (0.66 - 0.8)	0.57 (0.48 - 0.65)
**NPC2**	0.78 (0.66 - 0.9)	0.74 (0.66 - 0.82)	0.67 (0.59 - 0.76)	0.62 (0.55 - 0.7)
**Huang11**	0.75 (0.63 - 0.86)	0.63 (0.54 - 0.73)	0.73 (0.67 - 0.79)	0.55 (0.47 - 0.63)
**Gjoen7**	0.75 (0.61 - 0.88)	0.67 (0.59 - 0.76)	0.69 (0.6 - 0.77)	0.62 (0.55 - 0.69)
**Anderson38**	0.74 (0.63 - 0.85)	0.64 (0.55 - 0.74)	0.68 (0.6 - 0.76)	0.6 (0.52 - 0.68)
**Singhania20**	0.74 (0.6 - 0.87)	0.7 (0.63 - 0.77)	0.64 (0.56 - 0.72)	0.64 (0.56 - 0.72)
**Rajan5**	0.68 (0.56 - 0.81)	0.52 (0.42 - 0.62)	0.6 (0.52 - 0.68)	0.5 (0.43 - 0.58)
**Walter45**	0.47 (0.35 - 0.6)	0.66 (0.58 - 0.75)	0.54 (0.46 - 0.62)	0.49 (0.41 - 0.57)

Supplementary Table 10. Receiver operating characteristic (ROC) areas under the curve (AUC) (95% confidence intervals) showing diagnostic accuracy of candidate transcriptional signatures for incipient TB, stratified by time interval to disease, calculated in two-stage individual participant data meta-analysis as sensitivity analysis. ROC AUCs calculated for each contributing dataset; pooled estimates derived using random-effects meta-analysis. Number of samples included for each signature, at each time point, indicated in Supplementary Table 6.

**Signature**	**0 to 24**	**0 to 12**	**0 to 6**	**0 to 3**
**Suliman2**	0.8 (0.76 - 0.83)	0.85 (0.8 - 0.89)	0.96 (0.04 - 1)	0.89 (0.01 - 1)
**Kaforou25**	0.75 (0.65 - 0.82)	0.79 (0.72 - 0.84)	0.94 (0.18 - 1)	0.88 (0.79 - 0.93)
**BATF2**	0.75 (0.62 - 0.85)	0.78 (0.67 - 0.86)	0.87 (0.43 - 0.98)	0.89 (0.82 - 0.94)
**Roe3**	0.75 (0.55 - 0.88)	0.79 (0.62 - 0.89)	0.89 (0.39 - 0.99)	0.91 (0.79 - 0.97)
**Gliddon3**	0.73 (0.71 - 0.75)	0.77 (0.76 - 0.78)	0.9 (0.28 - 1)	0.85 (0.75 - 0.92)
**Sweeney3**	0.73 (0.64 - 0.81)	0.76 (0.72 - 0.79)	0.88 (0.33 - 0.99)	0.89 (0.8 - 0.94)
**Maertzdorf4**	0.71 (0.56 - 0.83)	0.74 (0.63 - 0.83)	0.86 (0.46 - 0.98)	0.85 (0.75 - 0.91)
**Suliman4**	0.71 (0.56 - 0.83)	0.72 (0.61 - 0.81)	0.87 (0.38 - 0.99)	0.89 (0.74 - 0.96)
**Gjoen7**	0.71 (0.56 - 0.82)	0.7 (0.63 - 0.77)	0.83 (0.41 - 0.97)	0.8 (0.56 - 0.92)
**Huang11**	0.68 (0.65 - 0.7)	0.7 (0.68 - 0.73)	0.66 (0.57 - 0.74)	0.82 (0.27 - 0.98)
**Zak16**	0.68 (0.64 - 0.71)	0.74 (0.73 - 0.75)	0.98 (0.07 - 1)	0.9 (0.58 - 0.98)
**Singhania20**	0.68 (0.6 - 0.74)	0.68 (0.6 - 0.75)	0.7 (0.58 - 0.8)	0.73 (0.42 - 0.91)
**NPC2**	0.68 (0.58 - 0.76)	0.7 (0.61 - 0.77)	0.83 (0.35 - 0.98)	0.72 (0.47 - 0.88)
**Anderson38**	0.67 (0.59 - 0.74)	0.69 (0.62 - 0.75)	0.82 (0.3 - 0.98)	0.75 (0.47 - 0.91)
**Qian17**	0.67 (0.55 - 0.76)	0.71 (0.6 - 0.81)	0.81 (0.4 - 0.97)	0.78 (0.75 - 0.8)
**Rajan5**	0.58 (0.42 - 0.73)	0.59 (0.38 - 0.77)	0.78 (0.1 - 0.99)	0.64 (0.25 - 0.91)
**Walter45**	0.55 (0.5 - 0.6)	0.57 (0.51 - 0.63)	0.57 (0.45 - 0.69)	0.54 (0.35 - 0.72)

Supplementary Figure 13. Diagnostic accuracy of eight best performing transcriptional signatures for incipient tuberculosis (TB) shown in receiver operating characteristic space, stratified by months to disease, calculated in two-stage individual participant data meta-analysis as sensitivity analysis (using bivariate random effects meta-analysis). Grey shaded zones indicate 95% confidence intervals for each signature. Cut-offs derived from two standard scores above the mean of control population. Dashed lines represent positive predictive value planes of 5, 10 and 10%, respectively, based on 2% pre-test probability. Number of samples included for each signature, at each time point, indicated in Supplementary Table 6.

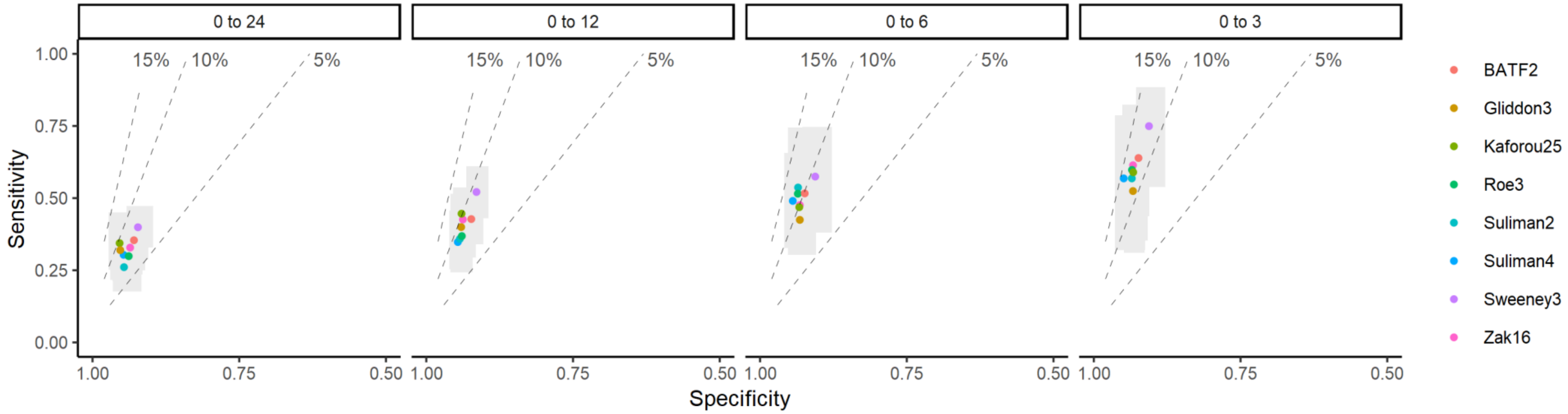

Supplement: Supplementary appendix 1 [file mmc1.pdf]
